# Improved atmospheric constraints on Southern Ocean CO_2_ exchange

**DOI:** 10.1073/pnas.2309333121

**Published:** 2024-01-30

**Authors:** Yuming Jin, Ralph F. Keeling, Britton B. Stephens, Matthew C. Long, Prabir K. Patra, Christian Rödenbeck, Eric J. Morgan, Eric A. Kort, Colm Sweeney

**Affiliations:** ^a^Geosciences Research Division, Scripps Institution of Oceanography, University of California, San Diego, La Jolla, CA 92093; ^b^Earth Observing Laboratory, National Center for Atmospheric Research, Boulder, CO 80307; ^c^Climate and Global Dynamics Laboratory, National Center for Atmospheric Research, Boulder, CO 80307; ^d^Research Institute for Global Change, Japan Agency for Marine-Earth Science and Technology, Yokohama 236-0001, Japan; ^e^Max Planck Institute for Biogeochemistry, Jena 07745, Germany; ^f^Department of Atmospheric, Oceanic and Space Sciences, University of Michigan, Ann Arbor, MI 48109; ^g^Global Monitoring Laboratory, National Oceanic and Atmospheric Administration, Boulder, CO 80309

**Keywords:** carbon sink, atmospheric diabatic mixing, inverse model, atmospheric transport model, airborne observation

## Abstract

We present improved estimates of air–sea CO_2_ exchange over three latitude bands of the Southern Ocean using atmospheric CO_2_ measurements from global airborne campaigns and an atmospheric 4-box inverse model based on a mass-indexed isentropic coordinate (M_θe_). These flux estimates show two features not clearly resolved in previous estimates based on inverting surface CO_2_ measurements: a weak winter-time outgassing in the polar region and a sharp phase transition of the seasonal flux cycles between polar/subpolar and subtropical regions. The estimates suggest much stronger summer-time uptake in the polar/subpolar regions than estimates derived through neural-network interpolation of pCO_2_ data obtained with profiling floats but somewhat weaker uptake than a recent study by Long et al. [*Science* **374**, 1275–1280 (2021)], who used the same airborne data and multiple atmospheric transport models (ATMs) to constrain surface fluxes. Our study also uses moist static energy (MSE) budgets from reanalyses to show that most ATMs tend to have excessive diabatic mixing (transport across moist isentrope, θ_e_, or M_θe_ surfaces) at high southern latitudes in the austral summer, which leads to biases in estimates of air–sea CO_2_ exchange. Furthermore, we show that the MSE-based constraint is consistent with an independent constraint on atmospheric mixing based on combining airborne and surface CO_2_ observations.

## Significance

Precise estimates of Southern Ocean (SO) CO_2_ uptake are lacking due to sparse surface–ocean observations. This study presents an alternate approach applying airborne CO_2_ observations to constrain the SO air–sea CO_2_ flux using a multibox atmospheric model aligned with moist isentropes. This study improves upon prior studies that estimate flux based on atmospheric CO_2_ measurements by using better-constrained estimates of atmospheric diabatic transport (transport across moist isentropes). It also allows fluxes to be resolved in finer latitude bands, thus facilitating a closer comparison with surface ocean pCO_2_ observations and identifying CO_2_ flux components driven by marine photosynthesis, ventilation, and warming/cooling. Our study underscores the value of aircraft measurements for precisely quantifying long-term changes in CO_2_ uptake by the SO.

Precise assessments of the air–sea CO_2_ flux of the Southern Ocean (SO), which includes both natural and anthropogenic components, are of critical importance to understanding the global carbon cycle and predicting future oceanic carbon uptake under climate change (1–4). The high-latitude SO (<58°S) was likely a significant natural source of CO_2_ to the atmosphere in the preindustrial era but has switched to being a net sink in the present-day (5). Available estimates suggest that uptake over the entire SO (<35°S) strengthened from 1980 to 2015, with significant decadal variability (4, 6–12).

Observation-based flux estimates of the entire SO remain highly uncertain. The net SO CO_2_ flux has been quantified using pCO_2_ measurements from ship-based and Argo float observations (7, 13–20) and from atmospheric CO_2_ measurements at surface stations that are inverted by atmospheric transport models (ATMs) (21–27). These products, however, show a large spread of flux estimates and are limited by sparse observations, possible measurement biases, and uncertainties in near-surface wind speed, gas exchange coefficients, and modeled atmospheric transport.

Recently, Long et al. [(28), henceforth Long21] used atmospheric CO_2_ observations from a series of global airborne campaigns to estimate the seasonal cycle of SO CO_2_ flux of a single region (90°S to 45°S) and reported an annual oceanic uptake of 0.53 ±   0.23 PgC y^−1^ averaged from 2009 to 2018. This annual sink estimate is consistent with the average of atmospheric inversion products (henceforth 3D inversions) and neural-network interpolation of ship-based pCO_2_ products (Surface Ocean CO_2_ Atlas, SOCAT) (15, 29) but larger than recent pCO_2_-based estimates using neural-network interpolation of profiling float data from the SO Carbon and Climate Observations and Modeling project (SOCCOM) (16, 17, 30). Long21 also identified a larger summer-time CO_2_ uptake compared to the SOCCOM-based flux estimates and the average of multiple atmospheric inversion products. The method of Long21 uses the atmospheric CO_2_ gradient across potential temperature (θ) as an emergent constraint on the underlying air–sea flux, taking advantage of the tendency of CO_2_ to be well-mixed on θ surfaces (31).

**Fig. 1.**
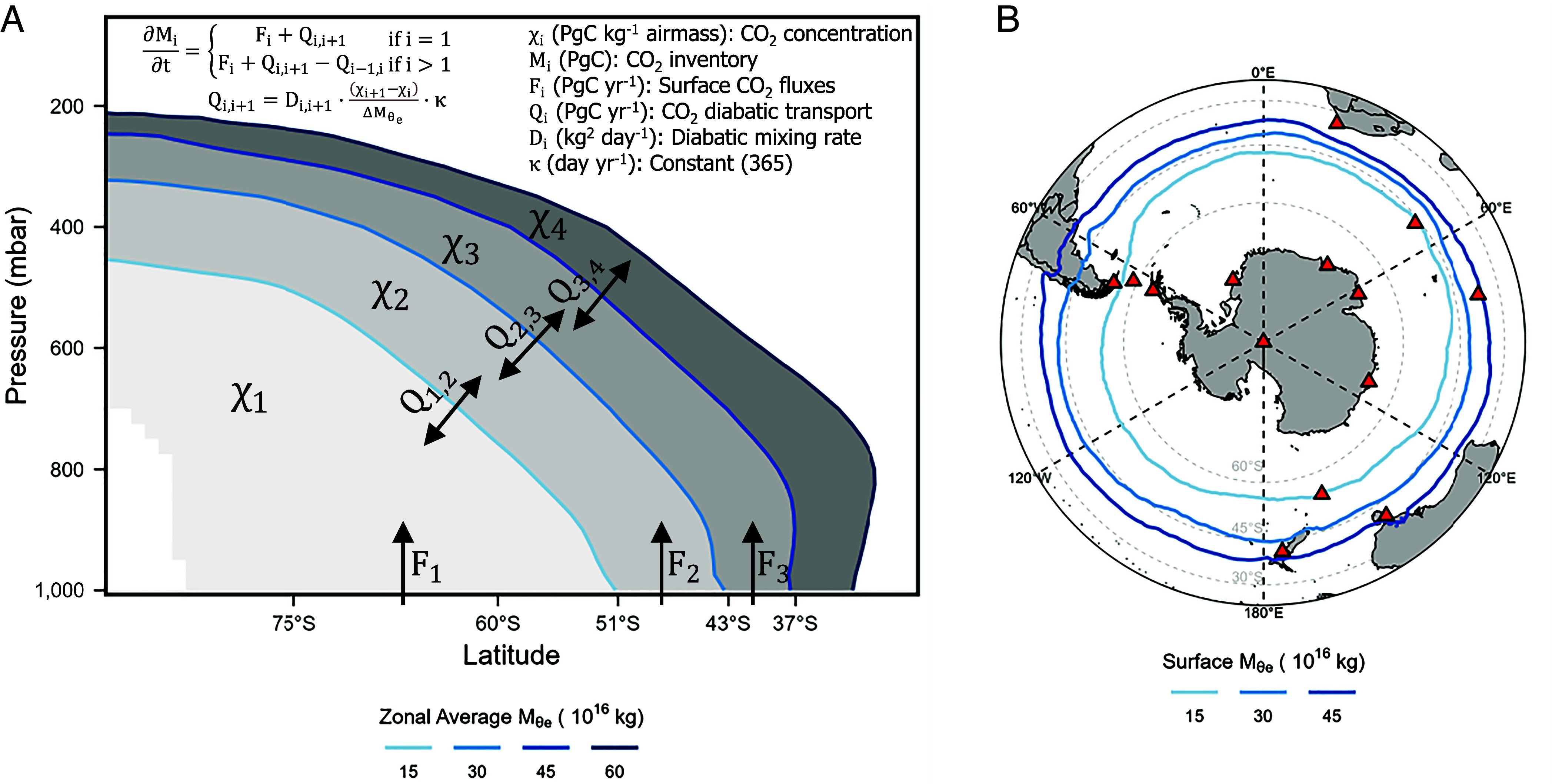
(*A*) Schematic of the box model. Boundaries of the box model are selected M_θe_ surfaces at 15, 30, 45, and 60 M_θe_ values (10^16^ kg), which are shown as zonal and 2009 to 2018 averages. (*B*) Selected near-surface M_θe_ contours as 2009 to 2018 averages. M_θe_ is computed from 3-h MERRA-2 reanalysis. These M_θe_ bands are nearly fixed with season (*SI Appendix*, Fig. S2). Red triangles show the location of surface stations that are used in the Carbon Tracker 2019b 3D (three-dimensional) CO_2_ inversion product.

Here, we provide improved estimates of seasonal SO CO_2_ flux using a 4-box tropospheric inverse method (Fig. 1*A*, henceforth 4-box inversion) and the same airborne datasets as in Long21 (detailed in *Materials and Methods* and *SI Appendix*, Fig. S1). Whereas Long21 resolved fluxes over a single domain (south of 45°S), our method resolves fluxes in three finer bands (“polar,” “subpolar,” and “subtropical”) between 90°S and ~37°S (Fig. 1*B* and *SI Appendix*, Fig. S2), which allows closer comparison with pCO_2_-based flux products (15–17) and provides insights into the latitudinal structure of processes driving seasonal pCO_2_ changes, such as the interactions between marine photosynthesis, ocean ventilation, and warming/cooling (32, 33).

At midlatitudes, CO_2_ and other long-lived tracers tend to be rapidly dispersed along surfaces of constant moist potential temperature (θ_e_), yielding gradients that are roughly parallel to the gradients in θ_e_ (34–37). Such mixing can be termed “adiabatic mixing,” in contrast to “diabatic mixing” which is defined as transport across θ_e_ surfaces involving diabatic heating or cooling. Our box model builds on recent work (38, 39) by aligning the box boundaries with fixed values of a mass-indexed isentropic coordinate M_θe_, which is parallel to θ_e_ at any instant time but is adjusted to conserve dry air mass in each box. This approach yields box boundaries that are nearly fixed with respect to latitude and season despite large seasonal displacements in θ_e_ and it highlights diabatic mixing as a critical process for quantifying large-scale tracer dispersion. Atmospheric transport is conventionally determined using ATMs, but these models show a large spread of simulated diabatic transport, which is related to uncertainty in advection, convection, and boundary height parameterizations (23, 40, 41). Prior studies have identified errors in ATMs by pointing to vertical CO_2_ gradients being overestimated in simulations at midlatitude (42, 43). We provide estimates of diabatic mixing rates that are independent of ATMs by using the moist static energy (MSE) budget of reanalyses. As MSE surfaces are identical to θ_e_ and M_θe_ surfaces, which are all conserved during adiabatic processes, MSE-based mixing rates provide precise constraints on cross-M_θe_ diabatic transport.

In this paper, we start by describing and validating the M_θe_-aligned box-model inversion method. We conduct a systematic analysis of uncertainty in ATMs-simulated diabatic mixing rates across three M_θe_ surfaces over the mid- to high latitude SO by developing two relevant constraints, one based on MSE budgets and the other based on atmospheric CO_2_ gradients across M_θe_ surfaces. We present our airborne-based seasonal flux estimates resolved from the box-model inversion method that is constrained by MSE-based diabatic mixing rates and discuss key features and mechanisms that cause the flux cycles to vary meridionally. Estimates obtained from airborne measurements are further compared with other flux products to identify any limitations these products may have. We also discuss the broad implications of our method for resolving decadal variability and long-term trends in SO CO_2_ fluxes, resolving surface fluxes of other species and in other regions, and the potential to improve ATMs in general.

## Results and Discussion

### Box-Model Architecture and Evaluation.

**Fig. 2.**
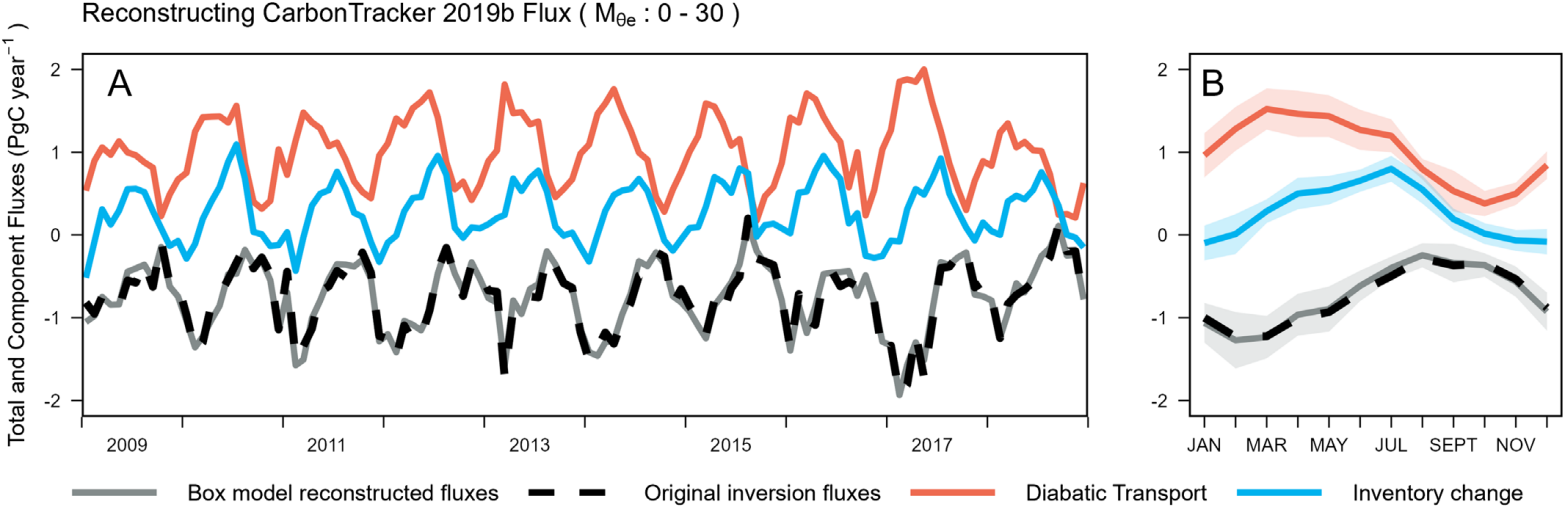
(*A*) Monthly reconstructed air–sea CO_2_ fluxes (solid gray) for the 0 to 30 (10^16^ kg) M_θe_ band (south of ~43°S near the Earth surface) based on CarbonTracker 2019b, compared with the original monthly 3D inversion fluxes for the same M_θe_ band (dashed black). The other components (i.e., diabatic CO_2_ transport and CO_2_ inventory change, detailed in *Materials and Methods*, and Eq. **1**) of the box-model reconstruction are shown as well. Positive values of the diabatic transport represent CO_2_ transport into the 0 to 30 M_θe_ band (poleward transport). We note that the inventory change (blue) equals the sum of fluxes (black) and diabatic transport (red). (*B*) Similar to (*A*), but showing the flux and other components as climatological monthly averages (2009 to 2018). Shaded regions show IAV, which is calculated as the SD over 10 y for the corresponding month. We also show these reconstructions for other 3D inversion products and other surface M_θe_ bands in *SI Appendix*, Figs. S4–S6.

The 4-box inversion model, shown in Fig. 1*A* (detailed in *Materials and Methods*), divides the troposphere in the Southern Hemisphere into discrete boxes, with lateral boundaries aligned with fixed values of M_θe_ (38). The M_θe_ coordinate is aligned with θ_e_, but a given M_θe_ surface constantly adjusts to keep the total dry airmass under it conserved. Each M_θe_ surface is indexed to the corresponding contained airmass. The three primary boxes of the model each contain 15 ×   10^16^ kg of dry air and intersect the surface of the Earth in zonal bands (Fig. 1*B*). The northernmost fourth box provides a boundary condition for the third box. The CO_2_ flux at the bottom of each primary box is calculated from mass balance, based on diagnosed CO_2_ transport between boxes and observed inventory changes within the boxes (Eq. **1**). A key assumption of the 4-box model is that the adiabatic transport (along θ_e_ or M_θe_ transport) is sufficiently rapid that CO_2_ meridional transport is mainly controlled by bidirectional diabatic transport (across θ_e_ or M_θe_ transport) between boxes, thus effectively reducing the troposphere to a discrete 1-dimensional mixing system. This assumption and the performance of the box model are validated below. In this model, diabatic transport is parameterized based on the cross-M_θe_ CO_2_ gradient and a seasonally dependent diabatic mixing rate, expressed in kg^2^ d^−1^ (Eq. **2**). Because airmass (kg) has replaced latitude or length in our box model, these mixing rates are analogous to diffusion coefficients, with the advantage of representing fundamental properties of the atmosphere that are independent of model discretization. We provide two approaches (*Materials and Methods*) to calculate climatological monthly diabatic mixing rates, one based on CO_2_ inversion systems that are constrained by surface CO_2_ observations and transport model simulations (ATM-based mixing rates) and one based on MSE budgets derived from MERRA-2 and JRA-55 reanalyses (MSE-based mixing rates).We validate the 4-box inversion approach by applying the method to reconstruct surface CO_2_ fluxes from four CO_2_ inverse models, using the full 3D gridded atmospheric CO_2_ fields of each product, averaged over each box, and using the corresponding parameterized climatological ATM-based mixing rates from the same model (*Materials and Methods*). This method provides an internally consistent system for each 3D inversion, and the reconstructed surface fluxes align well with original inverted fluxes over each zonal band (RMSE ≤ 0.12 PgC y^−1^, Fig. 2*A* and *SI Appendix*, Figs. S4–S6 and Table S1), especially over the climatological seasonal cycle (Fig. 2*B*). The 4-box inversion also reconstructs the interannual variability (IAV) of fluxes (e.g., Fig. 2*A*), even though the box-model uses interannually constant mixing rates, showing that flux IAV can be learned from variations in atmospheric CO_2_ gradients, while the impact of IAV on the atmospheric dynamics is relatively small. The method for resolving the zonal-averaged flux is not biased by the representation error (44, 45) that arises from the coarse resolution inverse model, which we verify by successfully reconstructing zonal-averaged air–sea CO_2_ flux from a product with finer-scale variability (*Materials and Methods* and *SI Appendix*, Fig. S16). These validations confirm that the complex 3D circulation of the atmosphere at high southern latitudes can be approximated by mixing along one dimension (the coordinate M_θe_), at least for the purpose of resolving zonal-averaged SO CO_2_ fluxes.

### Diabatic Mixing Rate Evaluation.

**Fig. 3.**
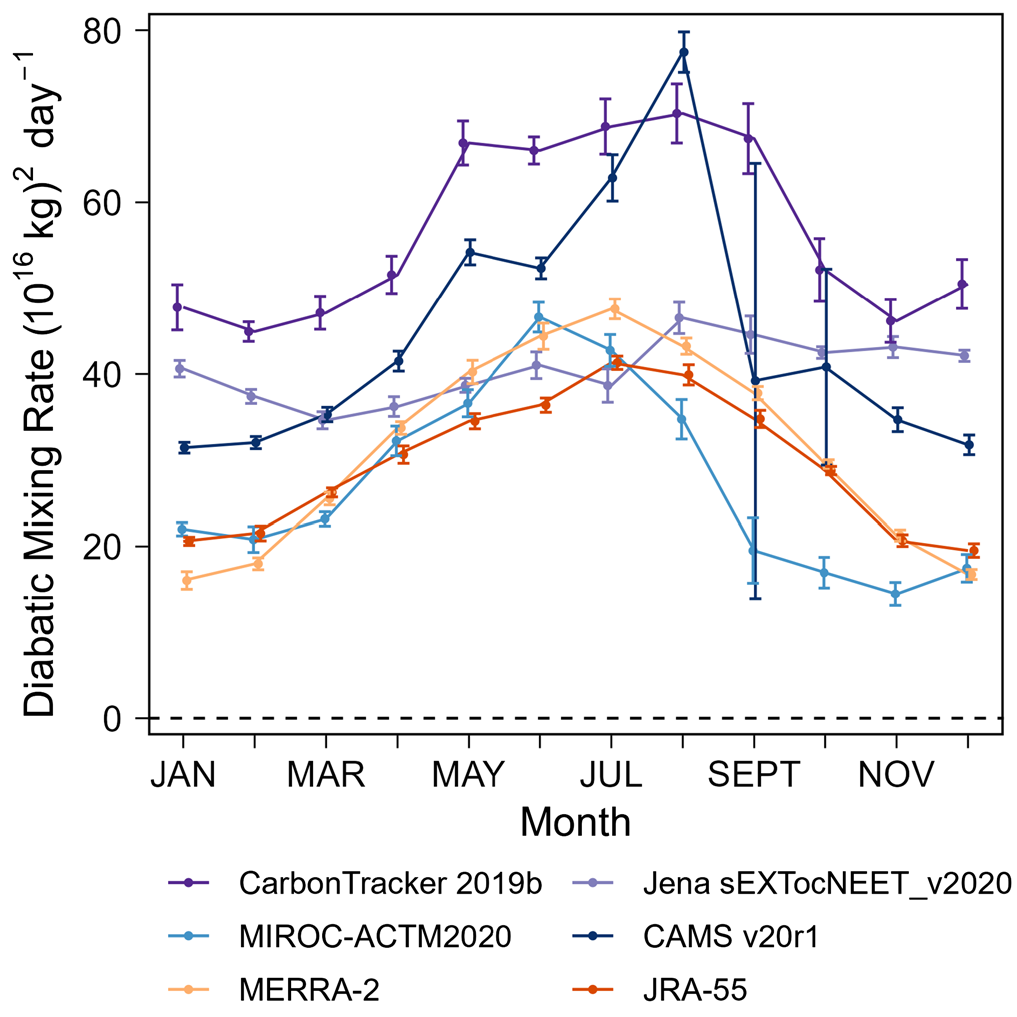
Diabatic mixing rates of the 30 (10^16^ kg) M_θe_ surface. These mixing rates are parameterized from four 3D CO_2_ inversion products and MSE budget of two reanalysis products (MERRA-2 and JRA-55). Error bars represent only the IAV of parameterized mixing rates, which is shown to be small, with the exception of CAMS in September because of the close-to-zero CO_2_ gradient across the 30 (10^16^ kg) M_θe_ surface. Diabatic mixing rates of the 15 and 45 (10^16^ kg) M_θe_ surface are shown in *SI Appendix*, Fig. S3.

**Fig. 4.**
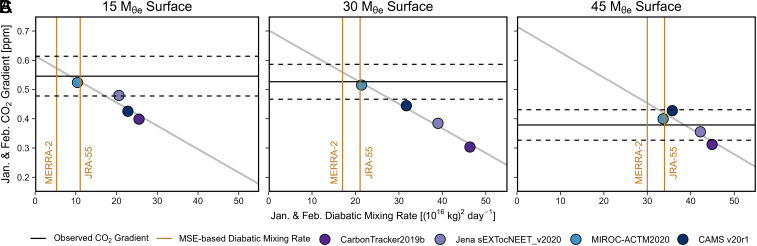
Exploring the correlation between January and February ATM-based mixing rates at (*A*) the 15 M_θe_ surface, (*B*) the 30 M_θe_ surface, and (*C*) the 45 M_θe_ surface and simulated atmospheric CO_2_ gradients across the corresponding M_θe_ surface of four transport models (3D CO_2_ inversion products). Simulated gradients are from 3D concentration fields averaged at the mean dates of five airborne campaigns or subcampaigns that took place during January and February (HIPPO1, ATom2, and ORCAS1-3). The corresponding ATM-based mixing rate is calculated as the January and February average. For comparison, we show the observed CO_2_ gradients (spatial bias corrected, as detailed in *SI Appendix*, Text S1) as horizontal black lines, which are calculated as the average of the same five campaigns or subcampaigns, while the dashed lines show the 1 σ uncertainty (measurement and spatial bias correction uncertainty). We also show two MSE-based mixing rates (January and February average) as vertical brown lines. A similar figure exploring the correlation between April to November averaged CO_2_ gradient and averaged diabatic mixing rate is presented in *SI Appendix*, Fig. S7.

We find that the MSE-based mixing rates from MERRA-2 and JRA-55 are highly consistent with each other, while ATM-based mixing rates have a large spread up to threefold and are faster than MSE-based mixing rates in austral summer over the high latitudes (Fig. 3 and *SI Appendix*, Fig. S3). We believe that the MSE-based mixing rates are more reliable for two reasons: First, the MSE-based constraint is powerful because surfaces of constant MSE are exactly parallel with the M_θe_ coordinate and because MSE has strong gradients across M_θe_ in all seasons. Second, the MSE-based constraint is consistent with an additional constraint on mixing that is available when combining CO_2_ data from both aircraft and surface stations. The available inverse models compute CO_2_ fluxes using surface data only but also yield troposphere CO_2_ gradients which can be compared to airborne observations. We find that the cross-M_θe_ CO_2_ gradients in most inverse models are inconsistent with the observed gradients in airborne data during the austral summer in the mid- to high latitude (Fig. 4 *A* and *B*). The discrepancies in simulated CO_2_ gradients correlate strongly with the diagnosed diabatic mixing rates from each corresponding ATM (Fig. 4), showing that ATMs with stronger diabatic mixing produce smaller CO_2_ gradients compared to observations. Based on the correlation, we find that the larger observed CO_2_ gradients from airborne data than model simulations appear to require a slower mixing rate at the 15 and 30 M_θe_ surfaces (Fig. 4 *A* and *B*), respectively, in the austral summer. The required mixing rates are consistent with the MSE-based mixing rate, thus providing strong evidence for the MSE-based estimates to be more realistic. Among all ATMs, the ACTM model yields a realistic summer gradient and mixing rates that are compatible with the MSE budget. In the rest of the year, both MSE-based mixing rates and ATM-based mixing rates, as well as simulated and observed CO_2_ gradients are generally within the 1 σ uncertainty of the observed gradients and close to two MSE-based mixing rates (*SI Appendix*, Fig. S7).

For the 4-box inversions presented here, we alternately use MSE-based mixing rates derived from MERRA-2 and JRA-55 to invert airborne CO_2_ observations, allowing for uncertainty in mixing based on the spread between these two estimates and their small IAV (detailed in *SI Appendix*, Text S2).

### Airborne-Based Air–Sea CO_2_ Fluxes.

We calculate air–sea CO_2_ fluxes using the observed CO_2_ inventory of each M_θe_ box and CO_2_ gradients across M_θe_ surfaces from each airborne campaign, which are resolved by binning airborne data into four M_θe_ bands (detailed in *Materials and Methods*). We correct for small biases in CO_2_ inventory and gradient induced by sparse spatial coverage of the airborne observations (*SI Appendix*, Text S1 and Tables S5 and S6) by comparing averaged CO_2_ from full 3D model data and flight track-subsampled model data. We also correct the contribution of small nonoceanic CO_2_ flux to the CO_2_ mass balance based on flux estimates in four inversion products (*SI Appendix*, Fig. S8). Our flux estimates allow for uncertainties from CO_2_ measurement imprecision, spread and IAV of MSE-based diabatic mixing rates, spatial coverage corrections, flux IAV due to insufficient temporal sampling, and nonoceanic CO_2_ flux corrections (*SI Appendix*, Texts S1 and S2). Although we report a similar random error as Long21, we expect our results to be subject to smaller systematic errors from uncertainty in atmospheric mixing and importantly also allow resolving fluxes at finer spatial scales with the same data. The reported random error is dominated by CO_2_ measurement error derived from comparing different instruments.

**Fig. 5.**
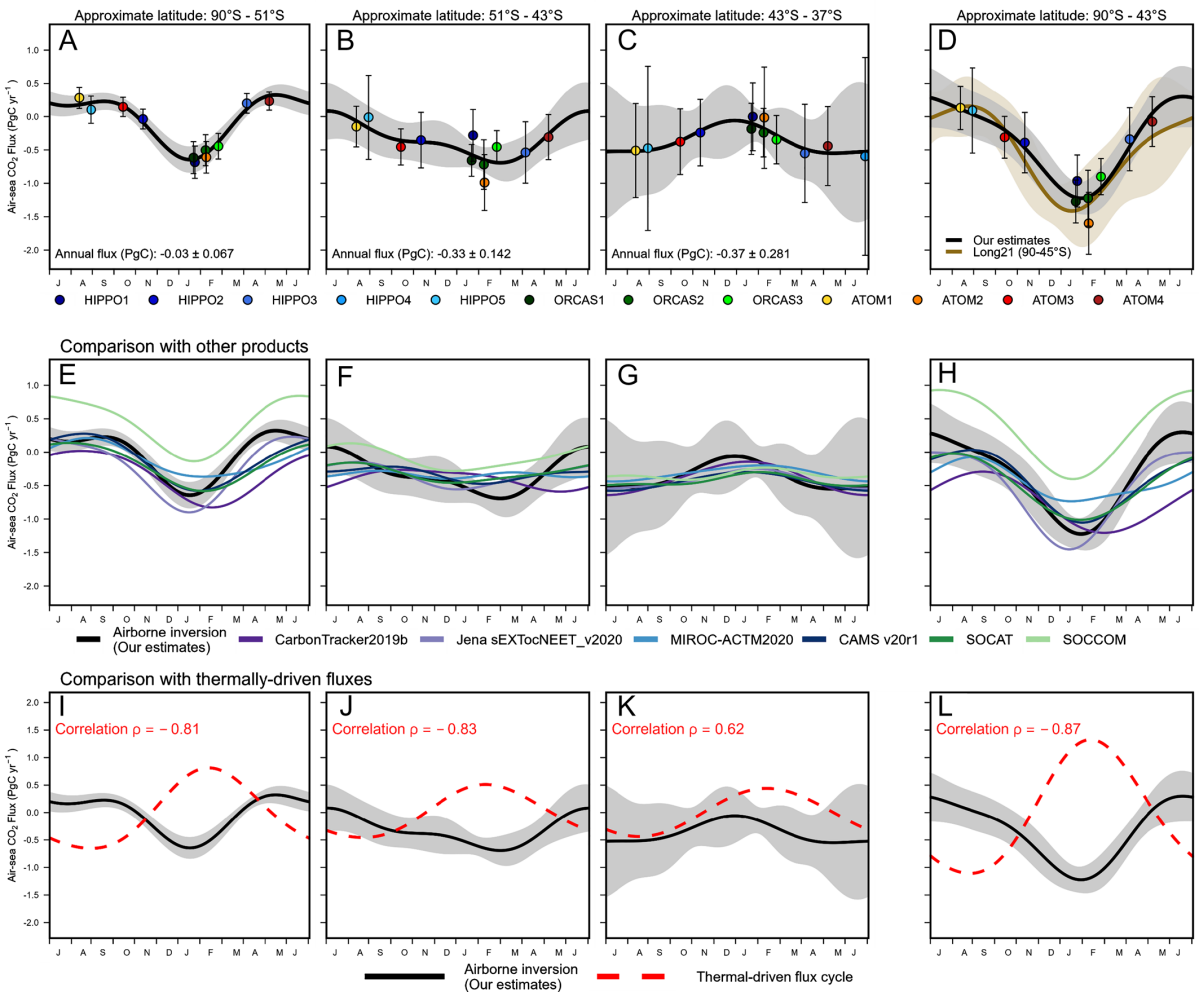
(*A*–*D*) Seasonal cycle of air–sea CO_2_ fluxes (negative as net oceanic uptake) estimated using the 4-box model based on airborne CO_2_ observations and two sets of MSE-based diabatic mixing rates (*Materials and Methods*). Each individual point represents the calculated fluxes using airborne observations from the corresponding campaign, centering on the mean date of each campaign, while the black line is a 2-harmonic fit. Error bars represent the 1 σ   uncertainty of each flux estimate, while shaded regions represent the 1 σ   uncertainty of the 2-harmonic fits (detailed in *SI Appendix*, Texts S1 and S2). Values of air–sea CO_2_ fluxes calculated for each airborne campaign transect and for each band are summarized in *SI Appendix*, Table S4. Annual fluxes are from the constant term of the 2-harmonic fitted climatological flux cycles, which is equivalent to integrating the fit over a year. These approximate latitude bands (see the *Top* of each panel) are calculated as the zonal average latitude of the corresponding annual average (2009 to 2018) M_θe_ surface over the ocean (*SI Appendix*, Fig. S2). We also show box-model resolved fluxes calculated using the average of six sets of mixing rates and each set of mixing rate in *SI Appendix*, Figs. S10 and S11. In (*E*–*H*), we compare our estimates with four 3D CO_2_ inversion products, and two neural network interpolated surface ocean pCO_2_ products using SOCAT pCO_2_ observations alone and SOCCOM pCO_2_ observations alone. Details of these products are in *SI Appendix*, Text S6. The SOCCOM product is a sensitivity run where all shipboard data from SOCAT were excluded (only SOCCOM float data were included). We note that the ocean CO_2_ flux in Jena sEXTocNEET_v2020 is a prior, which is provided by assimilation of surface ocean pCO_2_ observations (i.e., not neural-network derived pCO_2_) from SOCAT (29) by the Jena mixed-layer scheme (46). The seasonal cycle of each product is calculated as the average between 2009 and 2018, except for SOCCOM, which is averaged from 2015 to 2017. In (*I*–*L*), we compare our estimates with thermally driven air–sea CO_2_ flux cycles (dashed red, methods in *SI Appendix*, Text S3), which is derived from assuming 4% pCO_2_ increase per degree Celsius increase in sea-surface temperature (SST) and using wind speed–dependent gas exchange. We calculate the correlation between the airborne observed flux cycle and the estimated thermal-driven flux cycle of each band. Black solid curves and shaded regions in (*E*–*L*) are corresponding airborne observed fluxes and 1σ uncertainty. Panels (*I*–*L*) have a different *y*-axis range compared to panels (*A*–*H*). We also compare our estimates with nine global ocean biogeochemistry models that are used in the Global Carbon Budget 2020 (10, 47) in *SI Appendix*, Fig. S9.

The 4-box inversion resolves clear seasonal cycles of air–sea CO_2_ flux in all three latitude bands, with clear differences in amplitude and phasing between the bands. Over the polar band (Fig. 5*A*), we find a strong CO_2_ uptake in the summer (DJF) and a weak outgassing in the winter (JJA). Over the subpolar band (Fig. 5*B*), we find a strong uptake in the summer and a weak uptake in the winter. In the subtropical band (Fig. 5*C*), the seasonality is reversed, with a weak uptake in the summer and a strong uptake in the rest of the year. Averaged over the full year, all bands show net uptake. We now discuss each of these prominent features in turn.

The airborne-based estimates suggest a weak winter-time CO_2_ outgassing of 0.05 ±   0.03 PgC integrated from June to August (equivalent to 0.56 ±   0.35 gC m^−2^ mo^−1^) in the polar band (Fig. 5*A*). Winter outgassing is expected from strong winter-time upwelling which brings carbon-rich deep water to the surface (12). This outgassing pattern is consistent with several recent pCO_2_-based flux estimates, for example, observations from uncrewed surface vehicles in the Antarctic Zone during June and July of 2019 (0.7 gC m^−2^ mo^−1^) (48), reconstructed winter-time (July, 2004 to 2014 average) fluxes using summer-time measurements (0.04 ±   0.008 PgC) (49), and neural-network interpolation of ship-based SOCAT measurements (0.03 PgC, Fig. 5*E*) (15), but is smaller than estimates solely based on neural-network interpolation of SOCCOM float data during 2014 and 2017 (~0.23 PgC, Fig. 5*E*) (16). The small winter-time outgassing in our results is also consistent with several 3D inversions that used surface station CO_2_ observations (Jena inversion, ACTM, and CAMS) but is significantly more positive than one 3D inversion (CT 2019b, Fig. 5*E*). The airborne-based flux estimates show a clear phase shift between the polar/subpolar bands (Fig. 5 *A* and *B*) and the subtropical band (Fig. 5*C*). The boundary between these two boxes in the 4-box model roughly aligns with the subtropical front over the Atlantic and the Indian Ocean but is ~5°S of the subtropical front over the Pacific Ocean. This phase shift is likely due to the latitudinal change of the dominant mechanism that drives the surface–ocean pCO_2_ seasonal changes. To the north of this boundary, the pCO_2_ cycle is dominated by temperature-related solubility changes. To the south, it is dominated by biological production/mixing processes driving seasonal changes in dissolved inorganic carbon (32, 33, 50). A similar shift across ~40°S has been resolved in surface ocean pCO_2_ data (33, 50, 51) and also in flux estimates based on these pCO_2_ data, but the seasonal amplitudes of fluxes in these estimates are weaker in both regions than we find from airborne data (Fig. 5 *E*–*G*). The phase shift, however, is not distinctly resolved in the 3D inversions and two neural-network interpolations of pCO_2_-based products (Fig. 5 *E*–*G*). We note that inversions may be biased by excess diabatic transport in the austral summer (Fig. 4), and pCO_2_-based products are limited by sparse coverage, especially in our lowest latitude band 43 to 37°S (17).

To quantify the contribution of temperature-related solubility changes to the CO_2_ fluxes (Fig. 5 *I*–*L*), we compare the airborne-based fluxes to results from a simple thermal model, which assumes pCO_2_ increases by 4% per degree Celsius increase in sea-surface temperature (SST) change and uses wind-speed dependent gas exchange (methods in *SI Appendix*, Text S3) (52, 53). In the polar and subpolar zones (Fig. 5 *I*–*J*), the thermal model yields fluxes that are strongly out of phase compared with observations (correlation ρ = −0.81 and −0.83). In the subtropical region (Fig. 5*K*), the cycle from the thermal model broadly aligns with the observed cycle ( ρ = 0.62).

Despite the correlation, the observed flux cycle in the subtropical band has significant deviations in the austral spring compared to the thermal-driven cycle. The strengthening of CO_2_ uptake from January to April is faster than expected from warming alone (Fig. 5*K*), which requires a contribution from biological-driven changes, possibly associated with the fall phytoplankton bloom (54, 55).

We find a summer-time ocean CO_2_ uptake of 0.13 ± 0.04 PgC (integrated from December to February, DJF) in the polar band (Fig. 5*A*) and 0.14 ± 0.04 PgC in the subpolar band (Fig. 5*B*), which contributes to most of the annual uptake of 0.36 ± 0.16 PgC south of ~43°S (Fig. 5*D*). Our results are qualitatively consistent with prior estimates using the same airborne observations (Long21). However, our annual uptake estimate integrated over the polar and subpolar band is smaller (within uncertainty) than that of Long21 (0.53 ± 0.23 PgC). The difference is mainly explained by larger summer-time CO_2_ uptake in Long21, but the comparison is complicated by small differences in ocean domains between these two studies (the 30 M_θe_ surface, compared to 45°S, displaces ~2° southward over the western Pacific and ~3° in other basins). The larger summer uptake in Long21 can be attributed to the dependence on ATMs, which we suggest have unrealistically fast mixing rates in summer (Fig. 3). Summertime fluxes from our box model are especially sensitive to the diabatic mixing rate because summertime cross-M_θe_ gradients are large, and the inventory change is small (Fig. 2). The winter-time fluxes are less sensitive to the diabatic mixing rate because wintertime CO_2_ gradients are small, and the inverted flux is mainly diagnosed from the observed atmospheric CO_2_ inventory change.

Our results support prior work suggesting possible biases in SOCCOM pCO_2_ data (56). Compared to the airborne-based flux estimates, the SOCCOM-based estimates (Fig. 5 *E*–*H*) show significantly larger CO_2_ outgassing (or weaker uptake) all year round in the two high-latitude bands (Fig. 5 *E* and *F*). In these bands, the airborne-based results are in better agreement with flux estimates from SOCAT data.

Our airborne-based estimates show large differences from global ocean biogeochemistry models, which have known difficulties in representing CO_2_ exchange over the SO (7, 47, 57) given the large competing process drivers. We find several models that suggest similar phase shifts, but we did not find any model that agrees well with our estimates in all three bands (*SI Appendix*, Fig. S9). Airborne-based estimates are relatively consistent with pCO_2_-based estimates and inversions, while sharply deviating from GOBMs, underscoring the need for a better understanding of the physical and biogeochemical processes that drive SO air–sea CO_2_ fluxes in GOBMs.

### Overview and Outlook.

We have resolved air–sea CO_2_ fluxes over three zonal bands of the SO using airborne data and a 4-box inversion approach based on M_θe_ coordinates. This framework adequately describes large-scale CO_2_ transports needed for resolving fluxes at the scale of three zonal bands over the mid- to high latitudes of the SO, showing that the complex meridional CO_2_ transport can be simplified to diabatic transport. This framework also incorporates constraints on the diabatic mixing rate from MSE budgets of atmospheric reanalyses, without requiring an ATM. We demonstrate that the diabatic mixing rates inferred from the MSE budgets are realistic, based on a CO_2_ gradient-mixing rate constraint, but the mixing in most ATMs is too fast in the austral summer. These differences in representing mixing led to our summer uptake estimates being somewhat smaller than the uptake estimated by Long21, despite using the same airborne CO_2_ data. In the austral winter, ATM- and MSE-based mixing rates are generally comparable.

This study provides robust zonal average flux estimates from airborne data by capitalizing on rapid atmospheric mixing to integrate zonal heterogeneities. Our estimates have advantages over the published atmospheric inversions using surface station data because airborne data more accurately reflect large-scale features, and atmospheric vertical CO_2_ gradients are much more sensitive to fluxes than horizontal surface gradients. Also, our method is less sensitive to large uncertainties in simulated atmospheric mixing and the representation error due to model resolution (45). Compared to pCO_2_-based products, our estimates also have advantages, not being subject to uncertainty in gas exchange velocity and sparse coverage in pCO_2_ observations (28). A corresponding disadvantage, however, is the inability to resolve finer-scale spatial features.

The 4-box inverse model provides insights that have potential value for understanding and improving the simulated atmospheric circulation and structure in 3D ATMs. We show inconsistency in MSE-based and ATM-based diabatic mixing rates and in CO_2_ gradients between airborne data and inversion systems that are optimized by surface data (Fig. 4). These inconsistencies strongly motivate the incorporation of airborne data into CO_2_ inversion systems. They also identify key errors during the construction of modern ATMs related to diabatic mixing. Previous studies have highlighted uncertainty in vertical mixing as a major source of error in CO_2_ fluxes estimated via inverse model calculations (41, 42). Vertical mixing in the mid-troposphere has both along- and cross-M_θe_ components, and the cross-M_θe_ mixing (diabatic) component would typically be rate limiting because the along-M_θe_ (adiabatic) mixing is more rapid. Reducing uncertainty in vertical mixing thus requires reducing uncertainty in diabatic mixing, which we show can be constrained with MSE budgets. A first step would be to understand more fully the origin of the spread in mixing rates between ATMs. Based on the much larger spread in mixing rates between ATMs and the reanalysis products and the convergence of the MSE-based (from reanalysis) and ATMs-based mixing rates, we expect that the spread in ATMs mostly arises from different choices made in postprocessing of reanalysis data to generate ATMs, such as parameterization of convection or regridding and interpolation from the finer reanalysis grid to the coarser ATM grid. Future work should focus on ensuring that ATM mixing rates are consistent with the MSE budgets of the original reanalyses.

Our study motivates obtaining additional airborne data to improve estimates of large-scale carbon uptake across different latitudes of the SO. The ocean uptake over the entire SO has increased in recent decades according to surface ocean pCO_2_ data and models (1, 6–8, 10–12, 20). Here, we only attempted to resolve a seasonal climatology of the SO CO_2_ flux over different latitudes over the period 2009 to 2018, but resolving interannual variations would be feasible with regular sampling from Antarctic cargo aircraft. The M_θe_ coordinate is suitable also for studying the sources and sinks of other tracers, for example, computing air–sea O_2_ fluxes, and atmospheric CH_4_ chemical loss rates.

## Materials and Methods

### Airborne Campaigns and Airborne CO_2_ Observations.

We use airborne CO_2_ observations from three aircraft campaigns, the HIAPER Pole-to-Pole Observation project [HIPPO, (58), the O_2_/N_2_ Ratio and CO_2_ Airborne Southern Ocean Study [ORCAS, (59), and the Atmospheric Tomography Mission [ATom, (60). HIPPO and ATom have global coverage, mostly along a Pacific or Atlantic transect, while ORCAS focused on the SO adjacent to Drake Passage (horizontal flight tracks are shown in *SI Appendix*, Fig. S1). HIPPO consisted of five campaigns (HIPPO1 to 5) and ATom consisted of four campaigns (ATom1 to 4), each with several flights south of 35°S. ORCAS was a single 6-week campaign but with much denser temporal sampling, so we have split it into three subcampaigns (ORCAS1 to 3) in our analysis. Detailed descriptions of these airborne campaigns are in *SI Appendix*, Text S4 and Table S2. We primarily use CO_2_ airborne measurements collected by the NCAR AO2 instrument (61). To evaluate potential uncertainty (detailed in *SI Appendix*, Text S2.1), we also use measurements from three other in-situ instruments, the Harvard QCLS instrument (62), Harvard OMS instrument (63), and NOAA Picarro, and measurements from two flask samplers, the NCAR/Scripps Medusa flask sampler (61, 64) and NOAA Portable Flask Packages [PFP, (65)]. AO2 and QCLS are available on all campaigns. However, OMS did not fly on ORCAS or ATom, NOAA PFPs did not fly on ORCAS, and the NOAA Picarro did not fly on HIPPO. The in-situ measurements are averaged to 10-s intervals.

### Mass-Indexed Moist Isentropic Coordinate (M_θe_).

The M_θe_ coordinate, first introduced in the study by Jin et al. (38), is defined as the total dry air mass under a specific moist isentropic surface (θ_e_) in the troposphere of a given hemisphere. Surfaces of constant M_θe_ align with surfaces of constant θ_e_ but the relationship changes with season, as the atmosphere warms and cools. A schematic of the annual zonal average atmospheric M_θe_ value is in shown Fig. 1*A*, while climatological positions of the near-Earth surface contours of three M_θe_ surfaces (15, 30, and 45 10^16^ kg) are shown in Fig. 1*B* and *SI Appendix*, Fig. S2. Details of the calculation of M_θe_ are described in *SI Appendix*, Text S5.

We also relate bands of constant M_θe_ to approximate latitude bands (Fig. 5) based on the zonal average latitude of corresponding daily surface M_θe_ (averaged from 2009 to 2018) over the ocean.

### Box Model Architecture and Diabatic Mixing Rates.

We build a 4-box atmospheric model using selected M_θe_ surfaces (15, 30, 45, and 60, 10^16^ kg) as boundaries, shown in Fig. 1*A*. This box model takes advantage of θ_e_ (or M_θe_) being the preferential mixing surface of CO_2_ throughout the hemisphere, especially over midlatitude storm tracks (34, 37). The box model allows surface CO_2_ fluxes (F_i_, PgC y^−1^) to be computed from the CO_2_ mass balance of each M_θe_ box, based on the knowledge of atmospheric CO_2_ inventory ( $M_{i}$ , PgC) in each box and the diabatic transport of CO_2_ between boxes ( $Q_{i,i+1}$ , PgC y^−1^)

[1] $$\frac{\partial M_{i}}{\partial t}=\left\{ \begin{array}{l} F_{i}+Q_{i,i+1}\;\;\;\;\;\;\;\text{if}\;i=1\\ F_{i}+Q_{i,i+1}-Q_{i-1,i}\;\text{if}\;i<1 \end{array}\right.,$$

where i = 1 is the highest latitude (lowest M_θe_) box.

In Eq. **1**, $Q_{i,i+1}$ represents the transport (PgC y^−1^) of CO_2_ between the i^th^ and i + 1^th^ box, with poleward flux as positive. $Q_{i,i+1}$ is parameterized according to

[2] $$Q_{i,i+1}=D_{i,i+1}\cdot \frac{\left(\chi_{i+1}-\chi_{i}\right)}{\Delta M_{\theta_{e}}}\cdot \kappa ,$$

where $D_{i,i+1}$ is the diabatic mixing rate (kg^2^ d^−1^) that represents the mixing rate across the boundary of box i and i + 1, $\chi_{i}$ is the CO_2_ concentration (PgC per kg air mass) of the i^th^ box, calculated as CO_2_ inventory of the box divided by the total airmass of the box (15 × 10^16^ kg), and ∆M_θe_ is the distance in M_θe_ coordinates between box centers, which for evenly spaced boxes is the same as the total airmass of each box. κ is a constant (365) to convert from PgC d^−1^ to PgC y^−1^. Eq. **2** is a variant of Fick’s law, with M_θe_ as an effective distance coordinate, and $\frac{\left(\chi_{i+1}-\chi_{i}\right)}{∆{M_{\theta }}_{e}}$ is a measure of the CO_2_ concentration gradient. With this approach, $D_{i,i+1}$ is a property of the corresponding M_θe_ surface and is insensitive to the choice of box size.

We adopt two independent methods to estimate climatological (2009 to 2018 average) monthly diabatic mixing rates ( $D_{i,i+1}$ ). The first method extracts diabatic mixing rates from transport models using total CO_2_ fields from 3D inversion products (*SI Appendix*, Table S3). We first use the daily 3D atmospheric field of M_θe_ computed from MERRA-2 to assign a M_θe_ value to each daily model grid cell from 2009 to 2018. The atmospheric 3D CO_2_ fields and surface CO_2_ flux fields of inversions are interpolated to the MERRA-2 reanalysis grids (1° × 1°, 26 vertical levels from 1,000 to 100 mbar). We then calculate a daily CO_2_ inventory ( $M_{i}$ ) of each M_θe_ band as the sum of CO_2_ mass for all 3D grid boxes within the corresponding M_θe_ domain. We calculate monthly CO_2_ inventory change ( $\frac{dM_{i}}{dt}$ ) by taking the time derivative of the monthly atmospheric CO_2_ inventory. We note that monthly CO_2_ inventory change is computed by first averaging daily CO_2_ inventory by month but shifting the phase of the averaging window by 15 d to center at the beginning of each month and then differencing these values to obtain a rate of change centered midmonth. We calculate monthly CO_2_ gradients between two M_θe_ boxes ( $\chi_{i+1}$ − $\chi_{i}$ ) by averaging daily gradients. We calculate monthly surface CO_2_ flux ( $F_{i}$ ) by averaging daily flux, which is computed by integrating all daily 3D inversion flux grids with surface M_θe_ values within the corresponding M_θe_ range.

The CO_2_ transport across the north boundary of each M_θe_ box in the model can be calculated from the CO_2_ inventory change and surface flux of that box and the boxes further southward, according to

[3] $$Q_{i,i+1}\left(t\right)=\sum_{i^{\prime }=1}^{i^{\prime }=i}\left(\frac{dM_{i^{\prime }}\left(t\right)}{dt}-F_{i^{\prime }}\left(t\right)\right).$$

Combining Eqs. **2** and **3**, climatological average (2009 to 2018 average) monthly $D_{i,i+1}$ is calculated following

[4] $$D_{i,i+1}\left(t\right)=\frac{\left[\sum_{i^{\prime }=1}^{i^{\prime }=i}\left(\frac{dM_{i^{\prime }}\left(t\right)}{dt}-F_{i^{\prime }}\left(t\right)\right)\right]}{\left[\chi_{i+1}\left(t\right)-\chi_{i}\left(t\right)\right]}\cdot \Delta M_{\theta_{e}},$$

where [] denotes the average of corresponding monthly values of all years (2009 to 2018). The 1 σ uncertainty is calculated as the SD of resolved $D_{i,i+1}\left(t\right)$ for that month over all years, representing the IAV, which is shown to be small (Fig. 3 and *SI Appendix*, Fig. S3), with the exception of CAMS in September because of close-to-zero CO_2_ gradients across the 30 (10^16^ kg) M_θe_ surface.

The second method relies on MSE budgets from meteorological reanalyses, of which we use MERRA-2 and JRA-55 (66, 67). MSE is a measure of static energy that is conserved in adiabatic ascent/descent and during latent heat release due to condensation and is thus aligned with surfaces of θ_e_ or M_θe_. This method provides much more well-defined mixing rate estimates because finite MSE gradients exist in each reanalysis time step and do not reverse sign, in contrast to CO_2_. MSE is defined following

[5] $$MSE\left(t\right)=C_{p}\cdot T\left(t\right)+\text{gz}+L_{v}\left(T\right)q\left(t\right),$$

where $C_{p}$   (1005.7 J kg^−1^ K^−1^) is the specific heat of dry air at a constant pressure, T is temperature (K), g is the gravity constant assumed to be 9.81 ms^−2^, q is the specific humidity of air (kg water vapor per kg air mass), and $L_{v}$ is the latent heat of evaporation at temperature T (K). $L_{v}$ is defined as 2,406 kJ kg^−1^ at 40 °C and 2,501 kJ kg^−1^ at 0 °C and scales linearly with temperature.

MSE transport at the northern boundary of each box is calculated by energy conservation within the box, which follows Eq. **3** but has a small modification to account for atmospheric energy sources or sinks ( $E_{i}$ , J d^−1^):

[6] $$Q_{i,i+1}\left(t\right)=\sum_{i^{\prime }=1}^{i^{\prime }=i}\left(\frac{dS_{i^{\prime }}\left(t\right)}{dt}-F_{i^{\prime }}\left(t\right)-E_{i^{\prime }}\left(t\right)\right),$$

where S is the total MSE (J) that is calculated using temperature (T) and specific humidity (q) from corresponding reanalyses (Eq. **5**). $F_{i}$ is modified as surface heat flux (J d^−1^), including surface sensible and latent heat flux, which is directly available from MERRA-2 and JRA-55. $E_{i}$ is defined as heating rate due to radiative imbalance and is calculated using temperature tendency analysis ( $\frac{\partial T_{i}}{\partial t}$ , K d^−1^) of these reanalyses, following

[7] $$E_{i}\left(t\right)=C_{p}\left(T\right)\frac{\partial T_{i}\left(t\right)}{\partial t}M_{\theta_{e}}.$$

With MERRA-2, the temperature tendency due to radiative imbalance is directly available, while with JRA-55, it is calculated as the sum of heating rates due to longwave and shortwave radiation.

To estimate climatological monthly $D_{i,i+1}$ from reanalysis, the gradient ( $\chi_{i+1}-\chi_{i}$ ) in Eq. **4** is modified to be the energy density gradient (J per kg airmass), calculated from the total MSE of each box divided by the total airmass of the box (15 × 10^16^ kg in this study).

We thus calculate monthly $\frac{dS_{i^{\prime }}\left(t\right)}{dt}$ , $F_{i^{\prime }}\left(t\right)$ , $E_{i^{\prime }}\left(t\right)$ from 2009 to 2018 by averaging 6-hourly data from MERRA-2 and JRA-55, with 6-h S_i_ shifted by 15 d before calculating $\frac{dS_{i^{\prime }}\left(t\right)}{dt}$ , as for ATM CO_2_.

The calculation of monthly D based on MSE is according to a modified version of Eq. **4**:

[8] $$D_{i,i+1}\left(t\right)=\frac{\left[\sum_{i^{\prime }=1}^{i^{\prime }=i}\left(\frac{dS_{i^{\prime }}\left(t\right)}{dt}-F_{i^{\prime }}\left(t\right)-E_{i^{\prime }}\left(t\right)\right)\right]}{\left[\chi_{i+1}\left(t\right)-\chi_{i}\left(t\right)\right]}\cdot \Delta M_{\theta_{e}}.$$

We show six (four ATM-based and two MSE-based) sets of monthly diabatic mixing rates for the M_θe_ surfaces at 15, 30, and 45 (10^16^ kg) in Fig. 3 and *SI Appendix*, Fig. S3. Climatological daily mixing rates are further calculated by 4-harmonic fits to monthly data.

### Validation of the Box-Model Approach.

We validate the use of the 4-box model for estimating surface CO_2_ flux by showing that this approach successfully reconstructs monthly surface CO_2_ fluxes for each of the four 3D CO_2_ inversion products. This approach uses Eqs. **1** and **2**, with $\chi_{i}$ based on the gridded atmospheric CO_2_ fields averaged over grid cells within corresponding M_θe_ box and uses $D_{i,i+1}$ calculated using CO_2_ gradients from each transport model as described in the previous section. We then average daily reconstructed fluxes to monthly, centered at the middle of each month, shown as solid black curves in Fig. 2 and *SI Appendix*, Figs. S4–S6. We assess representation error due to the coarse resolution of the box model, by reconstructing the zonal-averaged flux from the neural-network interpolation of SOCAT data, using the 3D atmospheric field generated by the TM3 model with SOCAT-based air–sea CO_2_ flux, together with fossil fuel and ecosystem CO_2_ fluxes from the Jena sEXTocNEEv2020 (*SI Appendix*, Fig. S16). We find clear alignment between the original and reconstructed SOCAT-based flux, suggesting that our method is not limited by representation error.

### Airborne Estimates of Air–Sea CO_2_ Fluxes.

We use the 4-box model (Eqs. **1** and **2**) and airborne CO_2_ observations to calculate air–sea CO_2_ fluxes for each surface M_θe_ band and each airborne campaign, centering on the mean date of the campaign, shown as points in Fig. 5 *A*–*D*. This calculation includes the following steps.

We first detrend airborne CO_2_ observations by subtracting a smoothed interannual CO_2_ trend at the South Pole (SPO) (68). The trend is calculated by a stiff cubic spline function to the monthly average SPO data (69). We then compute the detrended average CO_2_ $\left(\hat{\chi_{i}}\right)$   for each campaign and each box by trapezoidal integration of detrended CO_2_ as a function of M_θe_ [as in the study by Jin et al. (38)] and dividing by the M_θe_ range of the box (i.e., 15 × 10^16^ kg). Prior to trapezoidal integration, we extrapolate airborne observations to M_θe_ = 0 surface using the average of the 100 observations with the lowest M_θe_ values near 0. The extrapolation only results in a slightly different averaged CO_2_ for the lowest M_θe_ box compared to the value without extrapolation (<0.03 ppm) because we have sufficient measurements across M_θe_ surfaces. The exceptions are HIPPO1 and 4 (difference ≈ 0.1 ppm), in which we do not have observations on low M_θe_ surfaces (*SI Appendix*, Fig. S15). For HIPPO, we only extrapolate airborne observations to the lowest M_θe_ values near 15 because due to the absence of observations in the entire first M_θe_ box, and only estimate fluxes for the 30 to 45 (10^16^ kg) box. We then correct for bias in CO_2_ estimates due to limited spatial coverage (detailed in *SI Appendix*, Text S1). For each M_θe_ box, we conduct a 2-harmonic fit with an annual offset to $\hat{\chi_{i}}$   of 12 campaigns, yielding a fitted seasonal cycle (with offset) of $\hat{\chi_{i}}$   . We then compute the long-term (2009 to 2018) time series of observed $\chi_{i}$   as the sum of the climatological seasonal cycle of $\hat{\chi_{i}}$   and the CO_2_ trend at SPO. We note that we use the same trend for each M_θe_ band, preserving each band’s annual mean offset from SPO. The time series of CO_2_ inventory ( $M_{i}$   ) of each box is therefore computed by multiplying $\chi_{i}$   and the M_θe_ range of the box (i.e., 15 × 10^16^ kg in this study). The fitted $\chi_{i}$   and $M_{i}$   values of each campaign are defined as the values at the mean date of the corresponding campaign. Observed surface CO_2_ fluxes for each airborne campaign are then calculated as the combination of two components, namely the CO_2_ inventory change $\frac{\partial M_{i}}{\partial t}$   and CO_2_ diabatic transport Q_i, i+1_, following Eqs. **1** and **2**. We calculate the component $\frac{\partial M_{i}}{\partial t}$ as the time derivative of the daily timeseries of M_i_ from combining the seasonal cycle fit and the SPO trend fit. The component Q_i, i+1_ for each airborne campaign mean date is calculated as the product of the observed atmospheric CO_2_ gradient (without fitting) between two boxes and the 4-harmonic fitted diabatic mixing rate at the campaign mean date (average of two MSE-based mixing rates) of the corresponding M_θe_ surface.

The surface CO_2_ fluxes estimated from the 4-box model are the total fluxes that also contain any land ecosystem CO_2_ emission/uptake and fossil fuel CO_2_ emission. We correct for these nonoceanic components by subtracting the corresponding flux components using the average of four 3D CO_2_ inversion products. The magnitude of this correction is small compared to the total air–sea fluxes, as shown in *SI Appendix*, Fig. S8.

We estimate the uncertainty of each individual flux estimate and the seasonal flux cycle by generating an ensemble (2,000 iterations) of flux estimates, allowing for uncertainty of these sources: 1) uncertainty of CO_2_ measurements; 2) uncertainty of the correction for spatial bias due to insufficient airborne coverage; 3) IAV of the diabatic mixing rate; 4) spread of the diabatic mixing rate between the two reanalyses; 5) correction for the biosphere and fossil fuel CO_2_ flux; and 6) IAV of the flux. Detailed bias and uncertainty analyses are presented in *SI Appendix*, Texts S1 and S2. The overall uncertainties of each flux estimate are shown as error bars in Fig. 5 *A*–*D*. The overall uncertainties of 2-harmonic fitted seasonal flux cycles are shown as shaded regions in Fig. 5 *A*–*D*.

We also show the averaged air–sea CO_2_ fluxes calculated using 6 sets of diabatic mixing rates (four sets of ATM-based and two sets of MSE-based) in *SI Appendix*, Fig. S10. These are estimated using the average and 1 σ uncertainty of 6,000 iterations of flux estimates, with 1,000 iterations for each set of mixing rates. We also show the air–sea CO_2_ fluxes calculated using each set of mixing rates in *SI Appendix*, Fig. S11.

We calculate the annual CO_2_ uptake of each M_θe_ box from the constant term of the 2-harmonic fitted seasonal flux cycles (shown as text in Fig. 5).

## Supplementary Material

Appendix 01 (PDF)

## Data Availability

The aircraft data are available in references for HIPPO (70), ORCAS (71), and ATom (72). All CO_2_ inversions are available via the University Corporation for Atmospheric Research/National Center for Atmospheric Research (UCAR/NCAR)—Digital Asset Services Hub Repository (73). Air–sea CO_2_ fluxes from neural-network interpolation of pCO_2_ products can be accessed from ref. 16. Air-sea CO_2_ fluxes from global ocean biogeochemistry models are available from ref. 74. MERRA2 reanalysis data are downloaded from the NASA Goddard Earth Sciences Data and Information Services Center at https://disc.gsfc.nasa.gov/datasets?project=MERRA-2. JRA-55 reanalysis data are downloaded from the NCAR Research Data Archive at https://rda.ucar.edu/datasets/ds628.0/dataaccess/.
