# Supplementary material for: Improved atmospheric constraints on Southern Ocean CO2 exchange
Source: Proc Natl Acad Sci U S A. 2024 Jan 30;121(6):e2309333121. doi: 10.1073/pnas.2309333121 (PMC10861854; doi:10.1073/pnas.2309333121)
Supplement: Supplementary file 1 — Appendix 01 (PDF) [file pnas.2309333121.sapp.pdf]

Supporting Information for

Improved Atmospheric Constraints on Southern Ocean CO_2 Exchange

Yuming Jin[1], Ralph F. Keeling[1], Britton B. Stephens[2], Matthew C. Long[2], Prabir K. Patra[3], Christian Rödenbeck[4], Eric J. Morgan[1], Eric A. Kort[5], Colm Sweeney[6]

[1]Scripps Institution of Oceanography, University of California, San Diego, La Jolla, California, USA

[2]National Center for Atmospheric Research, Boulder, Colorado, USA

[3]Research Institute for Global Change, Japan Agency for Marine-Earth Science and Technology, Yokohama, 236-0001, Japan

[4]Max Planck Institute for Biogeochemistry, Jena, Germany

[5]Department of Atmospheric, Oceanic and Space Sciences, University of Michigan, Ann Arbor, Michigan, USA

[6]National Oceanic and Atmospheric Administration, Earth System Research Laboratory, Boulder, Colorado, USA

Corresponding Author: Yuming Jin

Email: y2jin@ucsd.edu

This PDF file includes:

Supporting text S1 to S7

Figures S1 to S16

Tables S1 to S8

SI References

Supporting Information Text

Text S1: Bias due to sparse airborne spatial coverage

We showed in Fig. 3 and *SI Appendix*, Fig. S4-6 that, using the true 3-D atmospheric fields of models, we could successfully reconstruct the underlying surface CO_2 fluxes from knowledge of the true inventories of CO_2 within the $M_{\theta e}$ bands. Our estimated inventories from the airborne data may be biased, however, due to sparse coverage. To assess this bias, we compare the true χ_i of models (i.e., the 3-D atmospheric field of each inversion product) with χ_i calculated by subsampling the model atmospheric field along flight tracks. The true model χ_i is computed by averaging over all inversion product tropospheric grid cells (troposphere defined as potential vorticity unit, PVU, smaller than 2) of flight dates of each airborne campaign within the corresponding $M_{\theta e}$ box. The subsampled average χ_i is computed by subsampling model data along the flight tracks at flight dates and by trapezoidal integration of subsampled model data as a function of $M_{\theta e}$ (1), using $M_{\theta e}$ calculated from MERRA-2 and interpolated to the model grids. Prior to the trapezoidal integration, the subsampled data is also extrapolated to $M_{\theta e} = 0$ surface using the average of the 100 observations with the lowest $M_{\theta e}$ values, except for HIPPO4, in which we only extrapolate to $M_{\theta e} = 15$. We show the differences between true and subsampled averages in *SI Appendix*, Fig. S12 and Table S5 and S6. This comparison is conducted for each inversion posterior atmospheric CO_2 field, for each airborne campaign and for each $M_{\theta e}$ band. These four inversion products generally agree on the sign of the bias in CO_2 concentration due to sparse airborne spatial coverage. We correct our χ_i calculated from airborne observations for each $M_{\theta e}$ band and each airborne mission using the corresponding bias averaged over 4 inversion products. The 1σ uncertainty of the correction for each campaign (or sub-campaign) and $M_{\theta e}$ band is assumed to be the standard deviation of the corresponding corrections of four 3-D CO_2 inversions. The day-to-day variability (1σ) in model χ_i computed from the 3-D fields is small (< 0.05 ppm), thus this correction for sparse spatial sampling also effectively corrects for any temporal sampling biases from sampling on particular flight days.

Text S2: Uncertainty

We access the uncertainty of airborne-based seasonal air-sea CO_2 flux estimates of each $M_{\theta e}$ band by generating a large ensemble (2000 iterations) of flux estimates incorporating

uncertainty from the following sources: (1) uncertainty of airborne CO_2 measurements of instrument; (2) uncertainty of the bias correction for CO_2 concentration of each $M_{\theta e}$ band due to sparse airborne spatial coverage; (3) interannual variability of the diabatic mixing rates; (4) differences of diabatic mixing rates between two reanalyses; (5) uncertainty of correction for the biosphere and fossil fuel CO_2 flux; (6) interannual variability of air-sea CO_2 flux.

We first generate 2000 iterations of the airborne AO2 data that accounts for AO2 CO_2 measurement uncertainty (detailed below in *SI Appendix,* Text S2.1). For each iteration, we resolve detrended CO_2 for each $M_{\theta e}$ band and each airborne campaign or sub-campaign, while correcting for spatial bias with 1σ uncertainty of the correction incorporated (detailed in *SI Appendix,* Text S1). We then apply each iteration to the 4-box model to calculate surface CO_2 flux estimates for each $M_{\theta e}$ band and for each airborne campaign. We apply MERRA-based mixing rates to the first 1000 iterations and JRA-based mixing rates to the last 1000 iterations, with both sets incorporating interannual variability of the diabatic mixing rates as random errors (detailed in Materials and Methods). For individual flux (12 estimates) in each iteration, we add additional uncertainty due to flux interannual variability as suggested by MIROC-ACTM (details see below in *SI Appendix,* Text S2.2). Flux estimates from each campaign or sub-campaign and iteration are corrected for the small non-oceanic flux as the average of corresponding fluxes from four 3-D inversion models, while allowing 1σ uncertainty amounting to the standard deviation of four models (detailed in Materials and Methods). For each campaign, the overall 1σ uncertainty of flux (error bars in Fig. 5a-d) is calculated as the standard deviation of the 2000 iterations of flux estimates. We also calculate an ensemble of daily seasonal CO_2 flux cycles by carrying out 2-harmonic fits to each iteration of CO_2 flux estimates (12 campaigns or sub-campaigns) and for each $M_{\theta e}$ band. The 1σ uncertainty is calculated as the standard deviation of the 2-harmonic fitted daily flux of the large ensemble (2000 iterations), shown as shaded regions in Fig. 5a-d.

Text S2.1: Uncertainty of AO2 CO_2 measurement

The AO2 instrument is primarily an atmospheric oxygen instrument, which also includes a CO_2 sensor. Although this sensor is not as precise as the other sensors flown in these

campaigns, the short-term random error essentially averages out over the large spatial integrals used here. However, we allow that the AO2 CO_2 measurements may have systematic errors due to drift in calibration or other artifacts during or between flights. To address measurement error, we generate an ensemble (2000 iterations) of 10-sec airborne measurements (aligned with data in HIPPO, ORCAS, and ATom merged files), with each iteration representing a plausible representation of the AO2 CO_2 signal with error, following:

$$\mathrm{CO}_2^{i}(t) = \mathrm{CO}_2^{AO2}(t) + [\text{within flight error}] + [\text{between flight error}] \qquad \text{(S1)}$$

where $\mathrm{CO}_2^{i}(t)$ represents the ith iteration of CO_2, where i runs from 1 to 2000, and CO_2^{AO2} represents the original AO2 CO_2 data. Both within- and between-flight errors are estimated based on the differences in CO_2 (CO_2^{Diff}) measured between the AO2 instrument and other in-situ instruments (Harvard QCLS, Harvard OMS, or NOAA Picarro), assuming that the other measurements are correct and AO2 is wrong, which effectively provides a conservative assumption of errors in AO2. In this study, we estimate the error using AO2 and QCLS or OMS for HIPPO flights, and using AO2 and QCLS or NOAA Picarro for ORCAS and ATom flights.

The within-flight error is modeled as a random variable across all flights and measurements. For each flight and each pair of instruments (AO2 and the other), we build an autoregressive model using the method of Elorrieta et al. (2), which is suitable for irregular time series due to sampling gaps. The within-flight error is modeled as follows:

$$\mathrm{CO}_2^{Diff}(t) = \mathrm{AR}_{10-sec}^{j,k} \cdot \mathrm{CO}_2^{Diff}(t-1) + \epsilon^{j,k}(t) \qquad \text{(S2)}$$

where $\mathrm{AR}_{10-sec}^{j,k}$ is the autocorrelation coefficient that indicates the dependence of CO_2 of current time step $\mathrm{CO}_2^{Diff}(t)$ on that of previous time step $\mathrm{CO}_2^{Diff}(t-1)$, and $\epsilon^{j,k}(t)$ is the random error, drawn from a Gaussian distribution, with a new sample drawn for each data point. Both the AR coefficient and the standard deviation (1σ) of $\epsilon(t)$ are unique for each flight (j) and for each instrument pair (k), which we summarize in *SI Appendix*, Table S7. The 1σ random error is dominated by the short-term imprecision of the AO2 instrument, leading to an average uncertainty in CO_2^{Diff} of ±0.08 ppm. We note that the mean CO_2 offset of the flight (j) between two instruments is pre-subtracted while constructing the autoregressive model (Eq. S2), because between-flight error is considered separately. For

HIPPO flights, 1000 iterations are based on coefficients resulting from AO2 minus QCLS and 1000 iterations are based on AO2 minus OMS. For ORCAS and ATom flights, 1000 iterations are based on coefficients resulted from AO2 minus QCLS and 1000 iterations are based on AO2 minus NOAA Picarro. The order of these 2000 iterations is randomized for other error analyses. For flights where one of the instruments is unavailable (the target instrument AO2 and/or other instruments), we use the averaged AR coefficient and the averaged 1σ value for $\epsilon(t)$ of the corresponding campaign or sub-campaign and the corresponding instrument to generate simulated $CO_2^{Diff}(t)$.

The between-flight error is sampled from a Gaussian distribution centered on zero with a new sample drawn for each flight and applied as a uniform offset to all data in that flight. We use a standard deviation (1σ) of ± 0.26 ppm for all HIPPO flights, and ± 0.13 for all ATom and ORCAS flights based on AO2-QCLS differences. To establish these 1σ values, we compare the averaged CO_2 differences of each flight between AO2 and QCLS. The 1σ values are therefore calculated as the standard deviation of all flight-averaged CO_2 differences between two instruments, as shown in *SI Appendix,* Fig. S13. This approach gives a conservative estimate of AO2 flight-to-flight stability, as some variability could result from biases in the other sensor. Fig. S13 also shows differences between AO2 and other in-situ instruments (i.e., OMS and NOAA-Picarro) and NOAA portable flask packages (PFP) (3). Using ± 0.25 ppm for HIPPO based on AO2-OMS differences, ± 0.13 ppm for ORCAS and ATom based on AO2-NOAA Picarro differences, or ± 0.23 ppm for ORCAS and ATom based on AO2-PFP differences would not significantly change our results. The larger 1σ value for PFP comparisons might result from less data per flight with the PFP flask system. We did not compare with another flask dataset (Medusa) because the AO2 CO_2 measurements are already adjusted to match Medusa on a flight-average basis (4).

Text S2.2: Flux interannual variability (IAV)

In this study, we estimate 12 separate snapshots of the flux on particular dates that spread over 10 years, and fit a 2-harmonic seasonal flux cycle. Due to the interannual variability of the flux, our approximate seasonal cycle estimate will not conform to the true climatology. To estimate errors relative to a true climatology, we rely on IAV from inverted

oceanic CO_2 fluxes estimated using MIROC-ACTM. We access the flux bias due to limited temporal sampling for each airborne campaign (or sub-campaign) by comparing the ACTM modeled flux of a 15-day flight window and a 10-year averaged flux of the same 15-day window repeating from 2009 to 2018 (*SI Appendix,* Fig. S14). The 15-day flight window is selected as the mean campaign flight day ±7 days. We could correct for interannual variability, in principle, based on the difference between the modeled flux in a specific year and the 10-year averaged flux for each campaign. We find, however, only a small potential correction (bars in *SI Appendix,* Fig. S14, mean absolute correction of 0.04 PgC yr^{-1}). This bias also does not contribute to a clear seasonal flux cycle bias (black curves in *SI Appendix,* Fig. S14). Therefore, we do not correct for this bias but rather consider the flux IAV of each campaign (sub-campaign) as a random error, calculated as the standard deviation of the 15-day averaged flux from 2009 to 2018, as summarized in *SI Appendix,* Table S8. We use MIROC-ACTM to evaluate IAV because the simulated diabatic mixing rates and CO_2 gradients of ACTM match best with reanalysis and airborne observations.

Text S3: Thermal-driven CO_2 flux cycle

We estimate the thermal-driven flux cycle (Fig. 5i-l) using the following expression as suggested by Takahashi et al.(5).

$$F_{CO_2}^{thermal} = k \cdot \alpha \cdot pCO_2^{eq} \cdot 0.04 \cdot (SST - \langle SST \rangle) \quad (S3)$$

where k is the CO_2 gas transfer velocity (cm hr^{-1}), α is the CO_2 solubility in seawater (mmol m^{-3} atm^{-1}), pCO_2^{eq} is the CO_2 partial pressure at equilibrium, assumed to be 400 μatm, SST is the sea surface temperature, $\langle SST \rangle$ represents the annual average SST. 0.04 denotes a 4% pCO_2 change per 1°C SST change, also as suggested by Takahashi et al. (5).

We use monthly gridded (lon × lat = 1° × 1°) SST data from the NOAA Optimum Interpolation (OI) SST V2 product (6). The $\alpha \cdot pCO_2^{eq}$ term is a function of sea surface temperature (SST) and is calculated from CO2cal (7), by assuming salinity at 34 PSU, total alkalinity at 2250 μmol $kgSW^{-1}$, sea water density at 1.02 g cm^{-3}, and using monthly SST data from the NOAA OISST V2 product (6).

The gas exchange coefficient k is calculated following Wanninkhof, 1992 (8):

$$k = 0.31 \cdot U_{10}^2 \cdot \left(\frac{Sc}{660}\right)^{-\frac{1}{2}} \quad \text{(S4)}$$

where U_{10} is 10-m surface wind speed, which we obtained from MERRA-2 reanalysis (9), Sc is the Schmidt number, and 660 is the Schmidt number of CO_2 in seawater at 20°C. We calculate gridded monthly Sc from 2009 to 2018 using the expression below, as suggested in Wanninkhof, 1992 (8):

$$Sc = 2073.1 - 125.62 \cdot SST + 3.6276 \cdot SST^2 - 0.043219 \cdot SST^3 \quad \text{(S5)}$$

where we use monthly SST data from NOAA OISST V2 (6).

Text S4: Airborne campaigns

Both the HIPPO and ATom campaigns had broad coverage in the Southern Hemisphere, extending from the Equator to the Antarctic, and from near the surface (150-300 m) to the lower stratosphere (12-15 km) (*SI Appendix,* Fig. S1). HIPPO consisted of five missions (referred to as HIPPO1-5) between 2009 and 2011 and ATom consisted of four missions (refer to as ATom1-4) between 2016 and 2018 (detailed in *SI Appendix,* Table S2). HIPPO missions were over the Pacific Ocean, while ATom missions covered both the Pacific Ocean and the Atlantic Ocean.

ORCAS had 19 research flights during Jan. and Feb. of 2016, with spatial coverage from ~35°S to 75°S. These flights collected samples over the Drake Passage and surrounding South America and Antarctic Peninsula coastal regions. Since the ORCAS campaign spans about two months, we divide ORCAS flights into three groups (detailed in *SI Appendix,* Table S2) to yield the average CO_2 of each $M_{\theta e}$ band.

We exclude all observations near landing sites with the same criteria as in Jin et al. (2021), for example, samples that were collected 120s after takeoff, 600 s prior to landing, and likewise for any missed approaches.

Text S5: Calculation of $M_{\theta e}$ for each airborne observation

The computation of $M_{\theta e}$ is presented in Jin et al. (1), follows:

$$M_{\theta_e}(\theta_e, t) = \sum M_x(t)|_{\theta_{e_x} < \theta_e} \quad \text{(S6)}$$

where x indicates an individual grid cell of the atmospheric field, $M_x(t)$ is the dry air mass of each grid cell x at time t, and θ_{e_x} is the equivalent potential temperature of the grid cell.

For a given θ_e threshold, the corresponding $M_{\theta e}$ value is calculated by integrating the airmass of all grid cells with θ_e value smaller than the threshold. We only integrate airmass in the troposphere, which is defined as potential vorticity unit (PVU) smaller than 2. This calculation yields a unique value of $M_{\theta e}$ for each value of θ_e as well as a 3-D field of atmospheric $M_{\theta e}$, which we generate at daily resolution in the Southern Hemisphere based on MERRA-2 reanalysis (9). We also calculate $M_{\theta e}$ using other reanalyses (NCEP, JRA-55, and ERA-5) and we find that the differences are generally small (1).

We define surface $M_{\theta e}$ as the $M_{\theta e}$ value of the lowest available altitude level in the MERRA-2 reanalysis at a given longitude and latitude.

The $M_{\theta e}$ value of each airborne observation is computed by matching the observed θ_e value with our daily θ_e-$M_{\theta e}$ lookup table. We compute observed θ_e following

$$\theta_e = \left(T + \frac{L_v(T)}{C_p} q\right)\left(\frac{P_0}{P}\right)^{\frac{R_d}{C_p}} \quad \text{(S7)}$$

where T (K) is the temperature of air; q (kg of water vapor per kg of air mass) is the water vapor mixing ratio; R_d (287.04 J kg^{-1} K^{-1}) is the gas constant for air; C_{pd} (1005.7 J kg^{-1} K^{-1}) is the specific heat of dry air at constant pressure; P_0 (1013.25 mbar) is the reference pressure at the surface, and $L_v(T)$ is the latent heat of evaporation at temperature T. $L_v(T)$ is defined as 2406 kJ kg^{-1} at 40°C and 2501 kJ kg^{-1} at 0°C and scales linearly with temperature.

For HIPPO and ORCAS, we calculate θ_e using the recommended static pressure and air temperature variables (PSX and ATX), and water vapor mole fraction measured by VCSEL (10; H2Oppmv_vxl for HIPPO and VMR_VXL for ORCAS). We interpolate specific humidity in MERRA-2 to any missing water vapor mole fraction measurement along HIPPO flights. For ATom, we calculate θ_e from static pressure and air temperature as measured by the Meteorological Measurement System (MMS, P), and relative humidity of water vapor as measured by the Diode Laser Hygrometer (DLH, Sat_Vapor_Press_H2O) (11, 12).

Text S6: Atmospheric CO_2 inversion products, empirical surface ocean pCO_2 products, global biogeochemistry models, and prior airborne estimate

We use estimated air-sea CO_2 fluxes and posterior atmospheric CO_2 concentration from four atmospheric inversion products. The transport model, resolution, and meteorology of each inversion product are summarized in *SI Appendix*, Table S3. In these inversions, the fossil fuel and fire CO_2 fluxes are prescribed, while the ocean and land CO_2 fluxes are optimized to match in-situ CO_2 observations, except Jena sEXTocNEET_v2020 which uses prescribed ocean CO_2 fluxes provided by assimilation of surface ocean pCO_2 observations from SOCAT (13) by the Jena mixed-layer scheme (14). The land fluxes in Jena sEXTocNEET_v2020 are optimized.

Surface ocean pCO_2 products used for comparison purposes in this study were derived using neural-network approaches to interpolate pCO_2 data from SOCAT (13) or SOCCOM (15), as described in Landschützer et al. (16). Here we use two different flux estimates from Landschützer et al. (17, 18), with the methodology presented by Bushinsky et al. (19) and listed here by the names used in Fig. 5e-h:

1. SOCAT: only shipboard pCO_2 measurements were used to train the neural network and generate extrapolated pCO_2 fields used to estimate fluxes, no profiling float data were included.
2. SOCCOM(only): a sensitivity run where all shipboard data from SOCAT were excluded (only SOCCOM float data were included) south of 35°S after the year 2014.

We compare to modeled air-sea CO_2 flux fields from nine global ocean biogeochemistry models that were submitted to the Global Carbon Budget 2020 (20). These models are all general circulation models coupled with biogeochemistry modules. Details can be found in Hauck et al. (21) and Table A2 of Friedlingstein et al. (20). We downloaded flux fields from Hauck et al. (21), which have been previously regridded to 1° x 1°.

We use monthly surface $M_{\theta e}$ maps (averaged from the daily maps based on MERRA-2 that are interpolated to model grids to assign a $M_{\theta e}$ value to each surface grid of the CO_2 flux fields. Total fluxes of each month are calculated by integrating all selected grids of the corresponding $M_{\theta e}$ range. Seasonal cycles of total fluxes are calculated by a 2-harmonic fit

to the monthly fluxes from 2009 to 2018, except for SOCCOM and FESOM, which are from 2015 to 2017, and from 2009 to 2017, respectively.

For each product, we calculate annual uptake by integrating the monthly fluxes of each year, and we report the mean annual uptake from 2009 to 2018, with 1σ uncertainty as the standard deviation of 10 years.

Text S7: Discussion of diabatic mixing rates

We find that diabatic mixing rates are generally larger at a high $M_{\theta e}$ surface (lower latitudes) relative to a low surface (Fig. 2 and *SI Appendix*, Fig. S3), suggesting a faster diabatic transport time scale in low latitudes. Diabatic mixing rates also show a clear seasonal cycle at higher $M_{\theta e}$ surfaces (30 and 45), which display slower transport time scales (low values) in the austral summer relative to the winter. We show a large spread of mixing rates that are calculated from four different inversion products, corresponding to four different atmospheric transport models driven by four different reanalysis wind products (*SI Appendix*, Table S3). Among all four ATM-based mixing rates, CT-based mixing rates display the fastest transport, while ACTM-based mixing rates display the slowest transport. Jena-based mixing rates only show a small seasonal cycle and are close to ACTM-based mixing rates in the winter (small mixing rate), but relatively close to CT-based mixing rates in the summer (larger mixing rate). CAM-based mixing rates show a fast transport in the winter that is close to CT-based mixing rates, but show a slow transport in the summer that is close to ACTM-based mixing rates. On the other hand, mixing rates computed using moist static energy from reanalyses (MSE-based mixing rates) generally show a slower transport compared to the average of ATM-based mixing rates. The two MSE-based mixing rates are highly comparable and show a slow transport time scale that is close to ACTM-based mixing rates in the summer. Diabatic mixing rates only show very small interannual variability at each $M_{\theta e}$ surface and from each product, indicated by the small 1σ uncertainty bars in Fig. 2 and *SI Appendix*, Fig. S3. We note that ATM-based mixing rates are poorly constrained when the atmospheric CO_2 gradient across $M_{\theta e}$ is small (e.g., from September to November in CAMS). This suggests that, provided we use the same transport model, a different mixing rate would be derived if we base our calculations on a single component of atmospheric CO_2 (e.g., ocean flux alone), or if we use other chemical tracers (e.g., O_2) that have different gradients compared to CO_2.

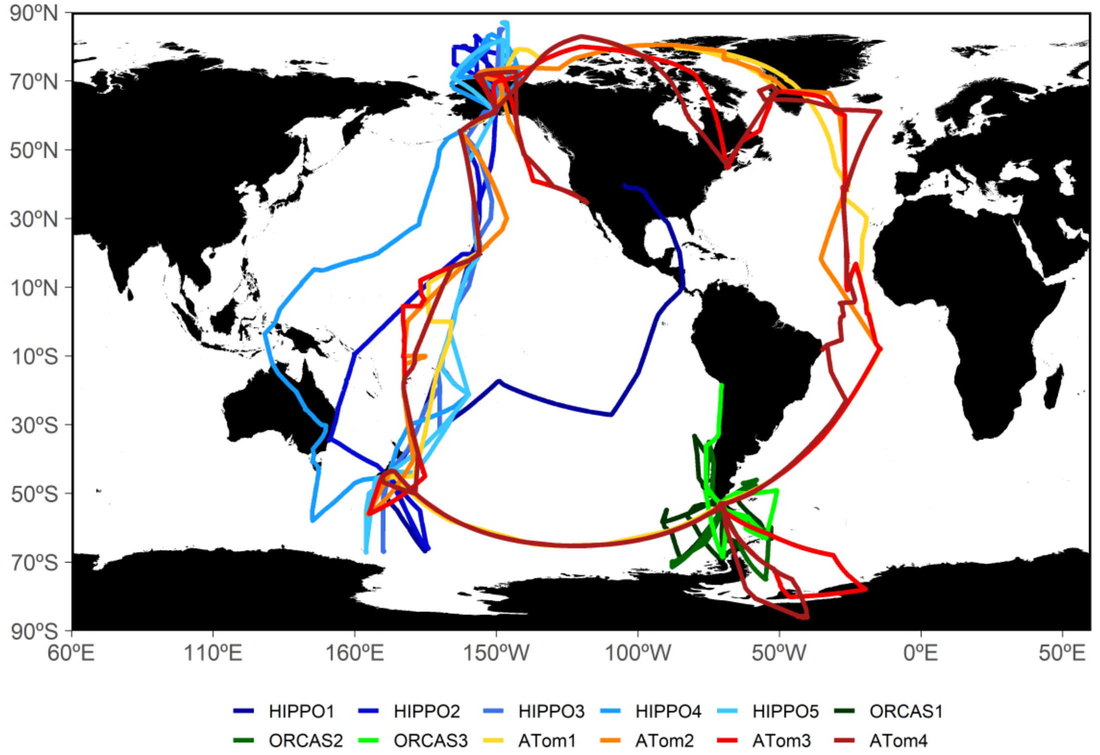

Fig. S1: HIPPO, ORCAS and ATom horizonal flight tracks, colored by campaigns or sub-campaigns. The aircraft profiled continuously from near the ocean surface to 12-14 km (see Fig. S1 in Long et al. 2021 (22)).

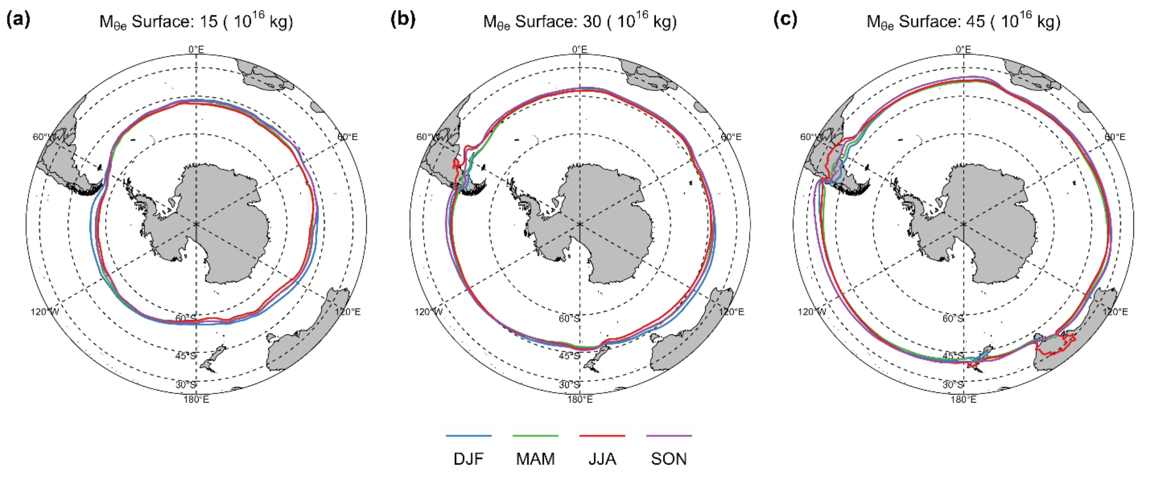

Fig. S2: Similar to Fig. 1b, but showing $M_{\theta e}$ surface contours for each season (by color, averaged from 2009 to 2018 based on MERRA-2 reanalysis) of three $M_{\theta e}$ surfaces (10^{16} kg).

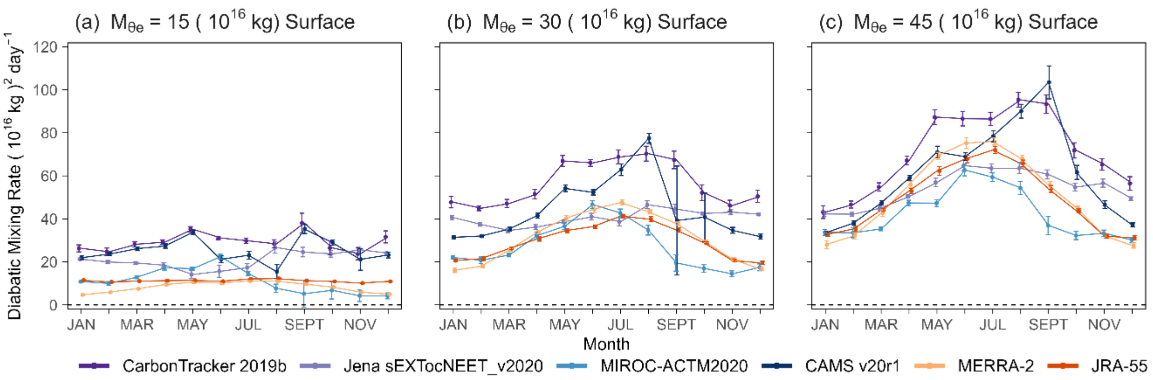

Fig. S3: Diabatic mixing rates of the (a) 15 (10^{16} kg), (b) 30 (10^{16} kg), and (c) 45 (10^{16} kg) $M_{\theta e}$ surface. These mixing rates are parameterized from four 3-D CO_2 inversion products and moist static energy budgets of two reanalysis products (MERRA-2 and JRA-55). Error bars represent only the interannual variability of parameterized mixing rates, which is shown to be small. Panel (b) is identical to Fig. 2, but with a larger y-range.

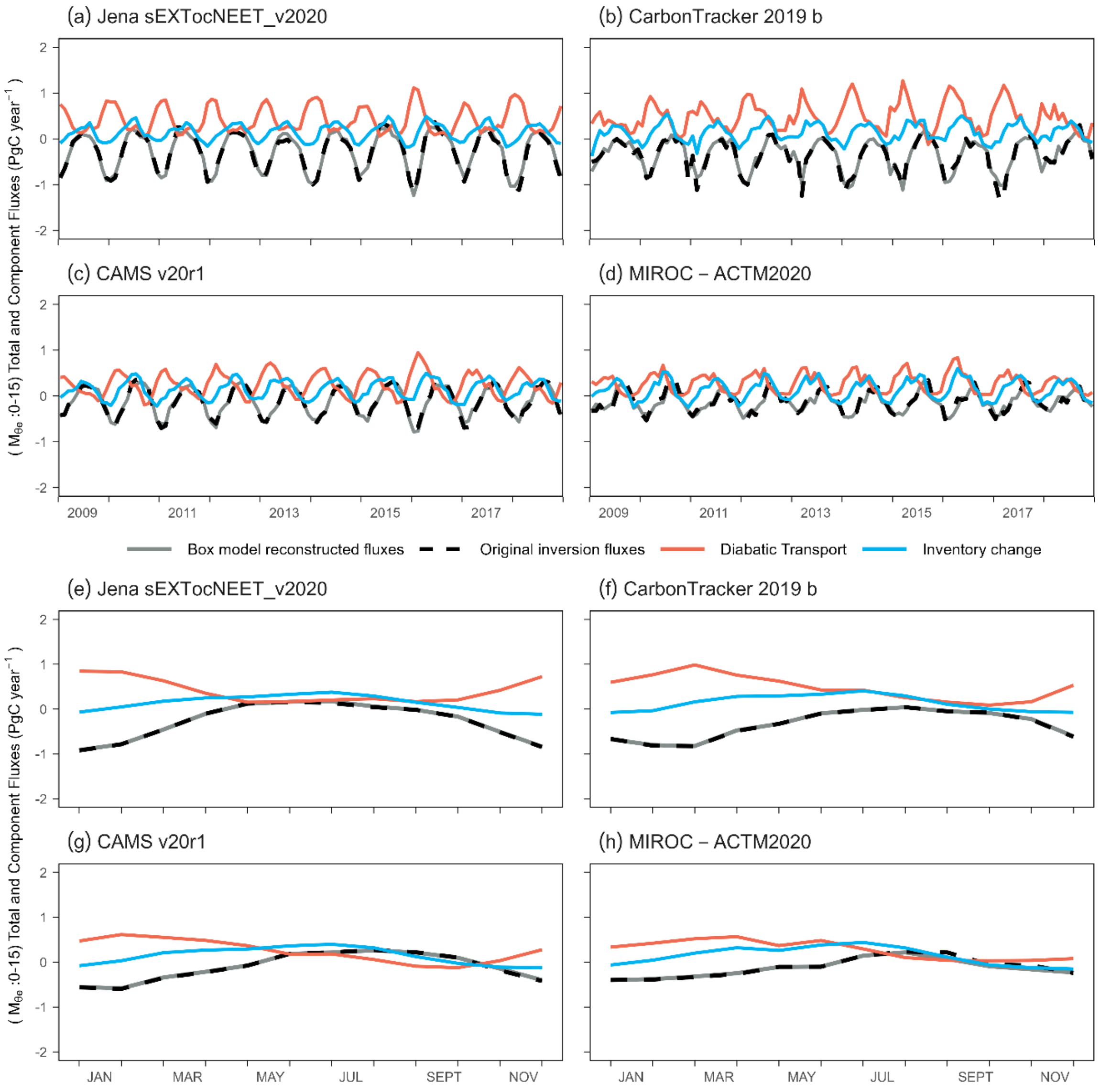

Fig. S4: (a) – (d) Monthly reconstructed air-sea CO_2 fluxes (solid black) of the 0-15 (10^{16} kg) $M_{\theta e}$ band (~ south of 51°S near the Earth surface, detailed in Fig. 1b and SI Appendix, Fig. S2) based on four 3-D inversions, comparing with the original monthly inversion fluxes of the same $M_{\theta e}$ band (dashed black). Each component (i.e., diabatic CO_2 transport and CO_2 inventory change, detailed in Materials and Methods, and Eq. 1) of the box-model reconstruction is shown as well. Negative values of the diabatic transport represents CO_2 transport into the 0-15 $M_{\theta e}$ band (poleward transport). (e) – (h) Similar to (a) – (d), but showing the flux and each component as a climatological monthly average (2009 to 2018).

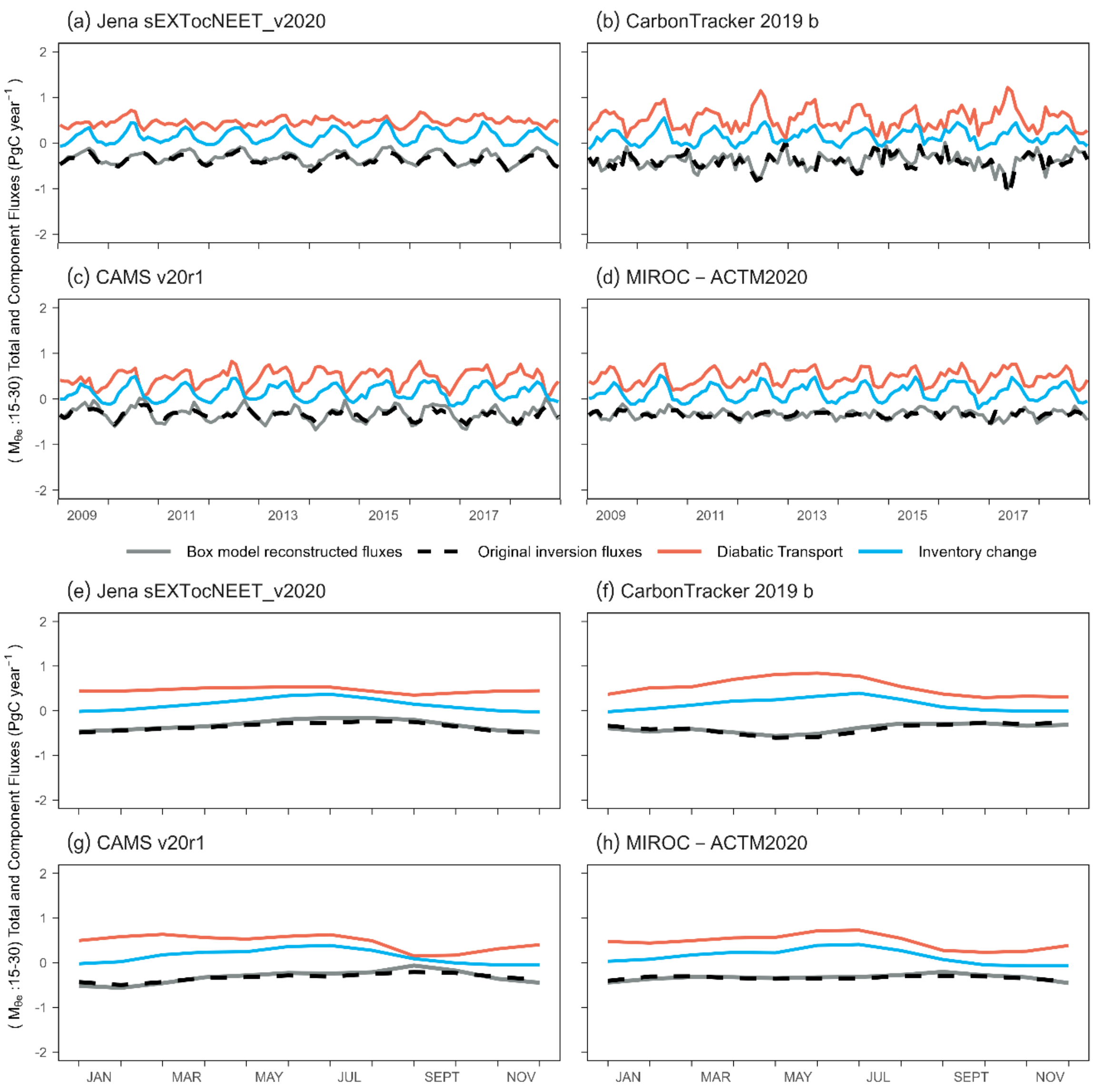

Fig. S5: Similar to Fig. S2, but showing reconstruction of surface CO_2 flux for the $M_{\theta e}$ band of 15 to 30 (10^{16} kg).

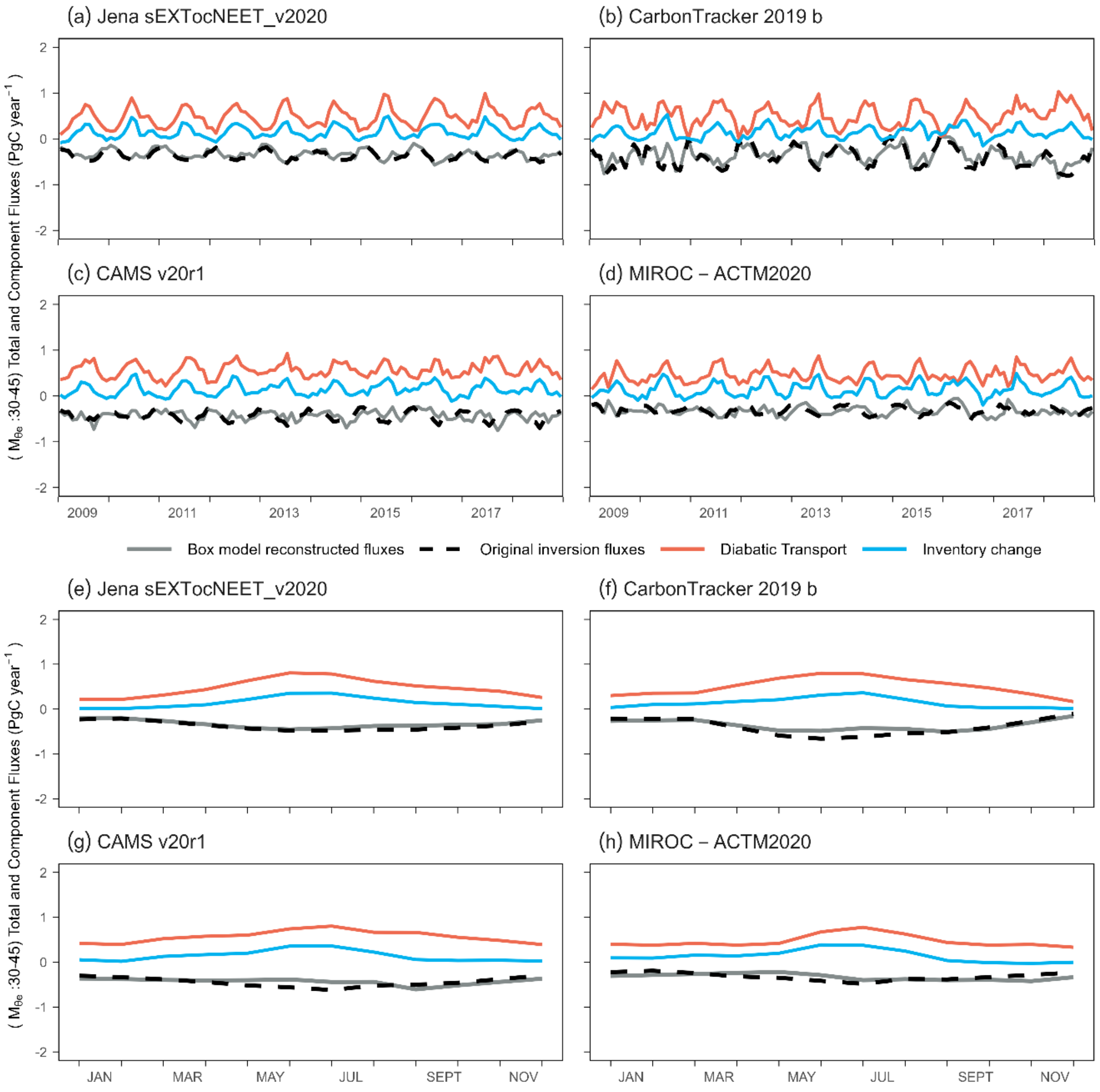

Fig. S6: Similar to Fig. S2, but showing reconstruction of surface CO_2 flux for the $M_{\theta e}$ band of 30 to 45 (10^{16} kg).

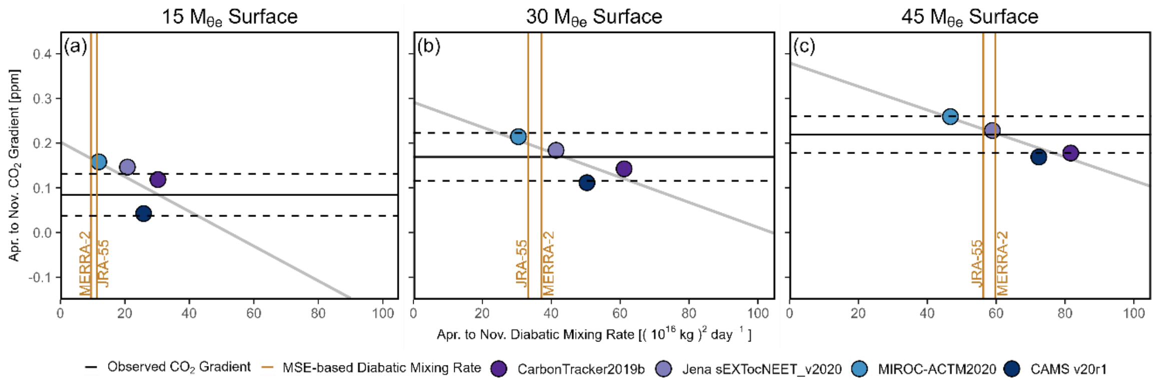

Fig. S7: Similar to Fig. 4a but exploring the correlation between April to November averaged ATM-based mixing rates for three $M_{\theta e}$ surfaces and simulated atmospheric CO_2 gradients across the corresponding $M_{\theta e}$ surfaces of four transport models (inversion products). Simulated gradients are averaged at the mean dates of seven airborne campaigns that took place during April to November (HIPPO2-5, and ATom1, 3, 4). The corresponding ATM-based mixing rate is calculated as the April to November average. For comparison, we show the observed CO_2 gradients (spatial bias corrected) in horizontal black lines, which are calculated as the average of the same seven campaigns, while the dashed lines show the 1 σ uncertainty (measurement and spatial bias correction uncertainty). We also show two MSE-based mixing rates (April to November average) as vertical brown lines.

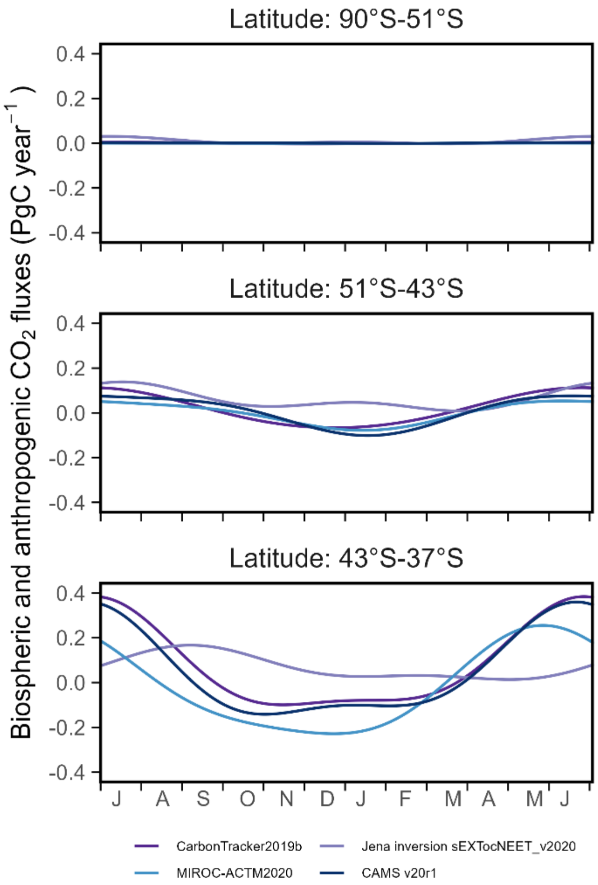

Fig. S8: Seasonal cycles (2009 to 2018 average) of biosphere and anthropogenic CO_2 fluxes estimated by the atmospheric inversion products for three approximate latitude bands (calculated based on surface $M_{\theta e}$ range). The seasonal cycles are calculated by 2-harmonic fits to monthly fluxes from 2009 to 2018. For each $M_{\theta e}$ band, we subtract the mean biospheric and anthropogenic flux (averaged from the four flux estimates) from our surface CO_2 flux estimates (based on airborne observation and box model) to yield air-sea CO_2 fluxes. This correction has 1σ uncertainty amounting to the standard deviation of the four flux estimates.

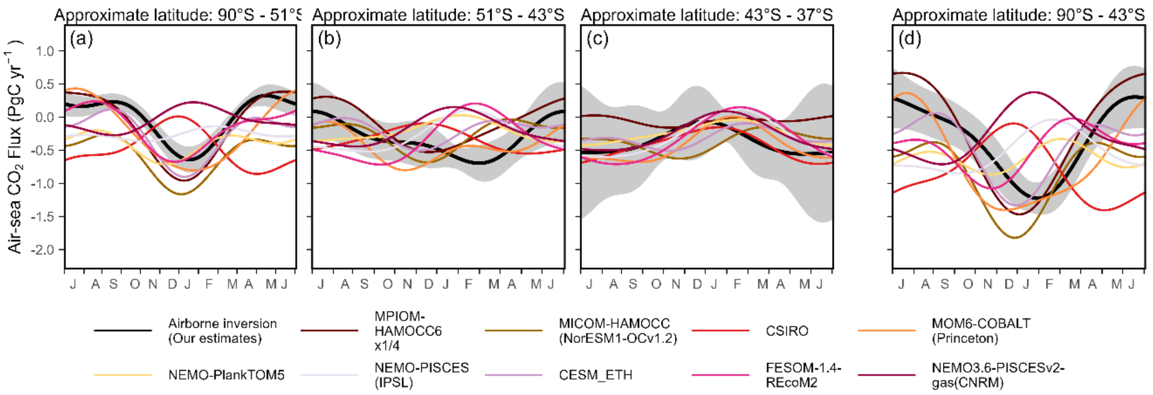

Fig. S9: Similar to Fig. 5e-h, but comparing our airborne-based estimates (black) with ocean biogeochemistry models that are used in Global Carbon Project 2020 (20, 21).

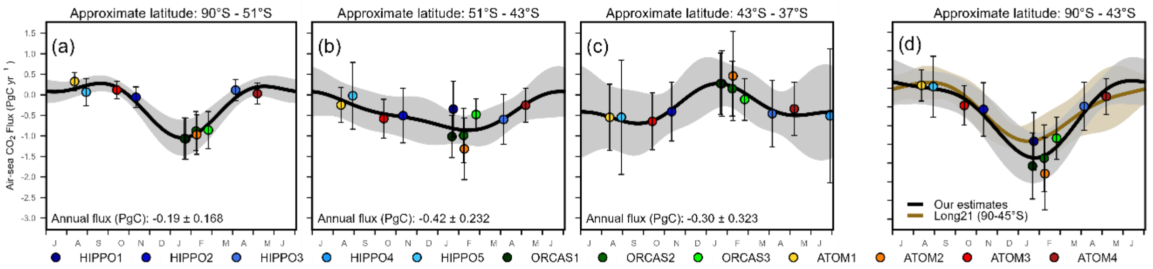

Fig. S10: Similar to Fig. 5a–d but showing the fitted flux cycles calculated using the mean of four ATM-based mixing rates and 2 MSE-based mixing rates.

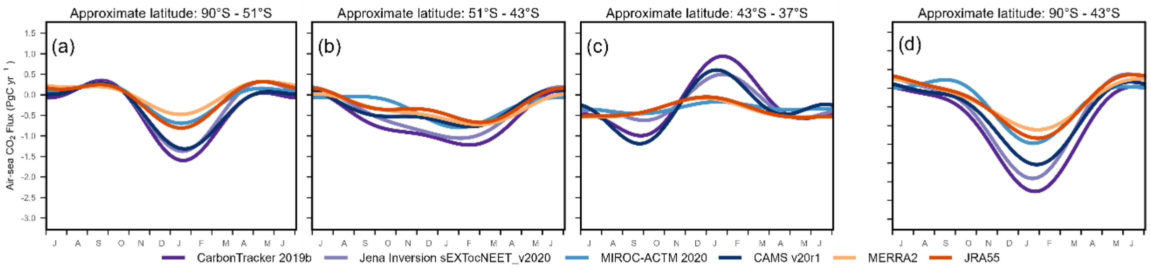

Fig. S11: Similar to Fig. 5a–d but showing the fitted flux cycles calculated using each set of diabatic mixing rate (i.e., 4 ATM-based and 2 MST-based).

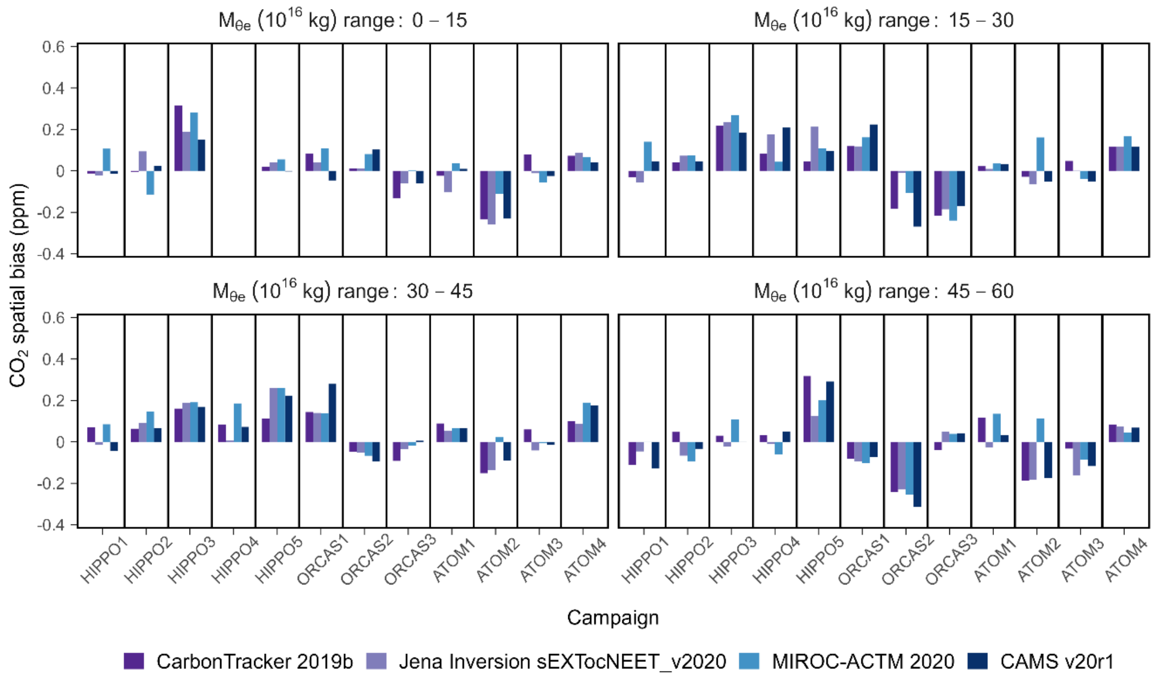

Fig. S12: Identifying bias in our estimates of CO_2 concentration for each $M_{\theta e}$ box due to limited spatial coverage of the airborne CO_2 measurements. We compare the true model CO_2 (i.e., calculated from the 3-D atmospheric field of each inversion product) with values calculated by subsampling the model atmospheric field along the flight track of each airborne mission (method see *SI Appendix,* Text S1) and processing identically to the observations. The bias is calculated as the subsampled average minus the true average, and therefore, a positive bias indicates that the limited spatial coverage biases the estimated CO_2 concentration too large. We adjust our measurements using the average across models for each campaign or sub-campaign.

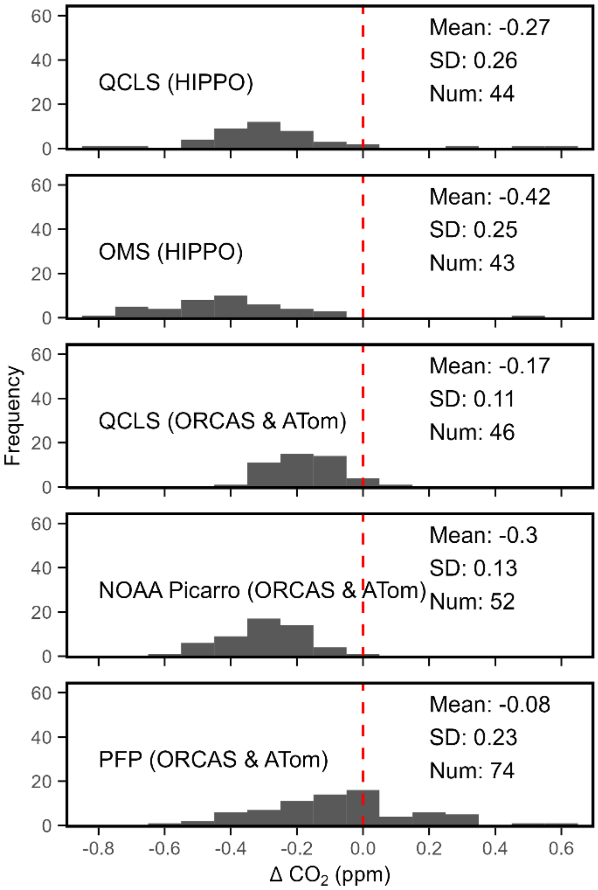

Fig. S13: Histogram of CO_2 differences, averaged over each flight, between the AO2 instrument and other instruments (for method see *SI Appendix,* Text S2.1). One value is generated per flight and the histogram shows these differences across all flights and campaigns. We also show the mean and standard deviation of CO_2 offsets for each instrument, and the number of flights that are available.

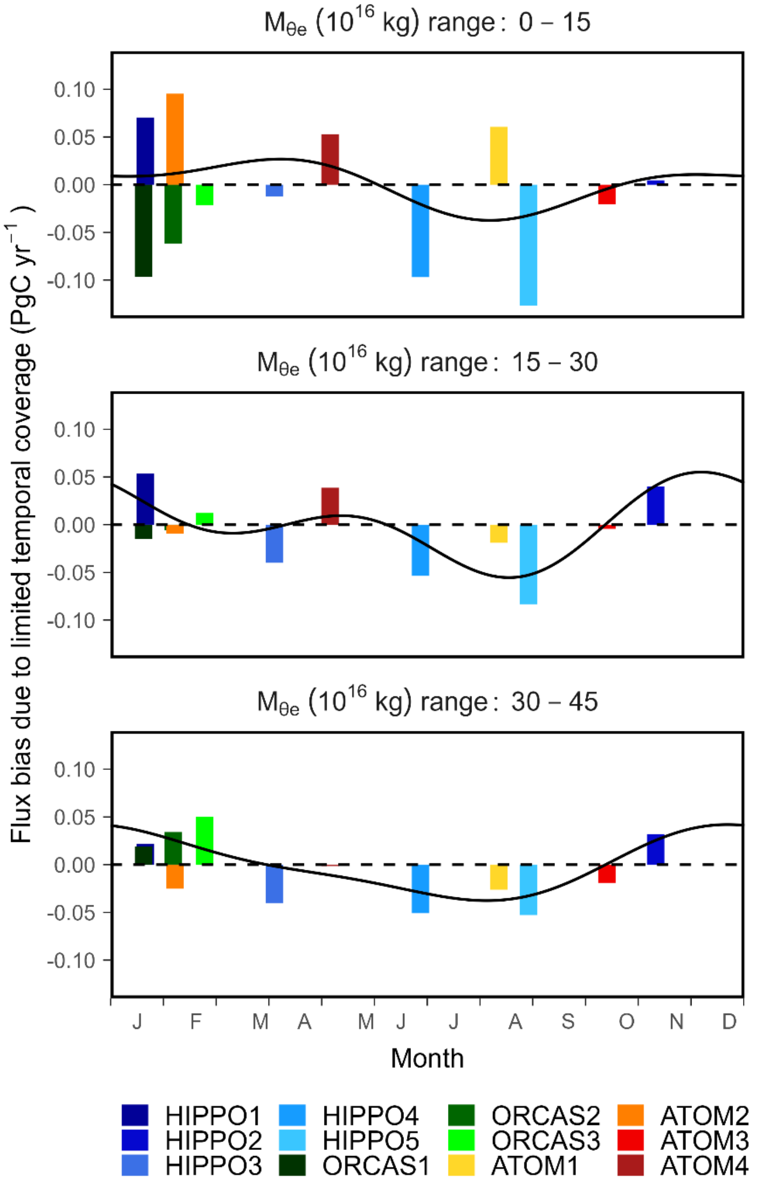

Fig. S14: Identifying bias in CO_2 flux estimates for each $M_{\theta e}$ box and each airborne campaign or sub-campaign due to limited temporal coverage (interannual variability), based on estimated flux from MIROC-ACTM. For each campaign, we quantify the bias (shown as bars) as the differences between the modelled flux of a 15-day flight window around the corresponding campaign mean date and the 10-year averaged flux of the same 15-day window repeating from 2009 to 2018. The 15-day flight window is selected as the mean campaign flight day ±7 days. A positive bias indicates that the limited temporal coverage biases the estimated air-sea CO_2 flux too large (more outgassing or less uptake). We also show the corresponding seasonal cycle of these interannual flux biases as black curves, estimated by 2-harmonic fits of corresponding bars for each $M_{\theta e}$ band. We do not adjust for interannual sampling biases, but do include a component in our uncertainty budget from inverted flux in MIROC-ACTM.

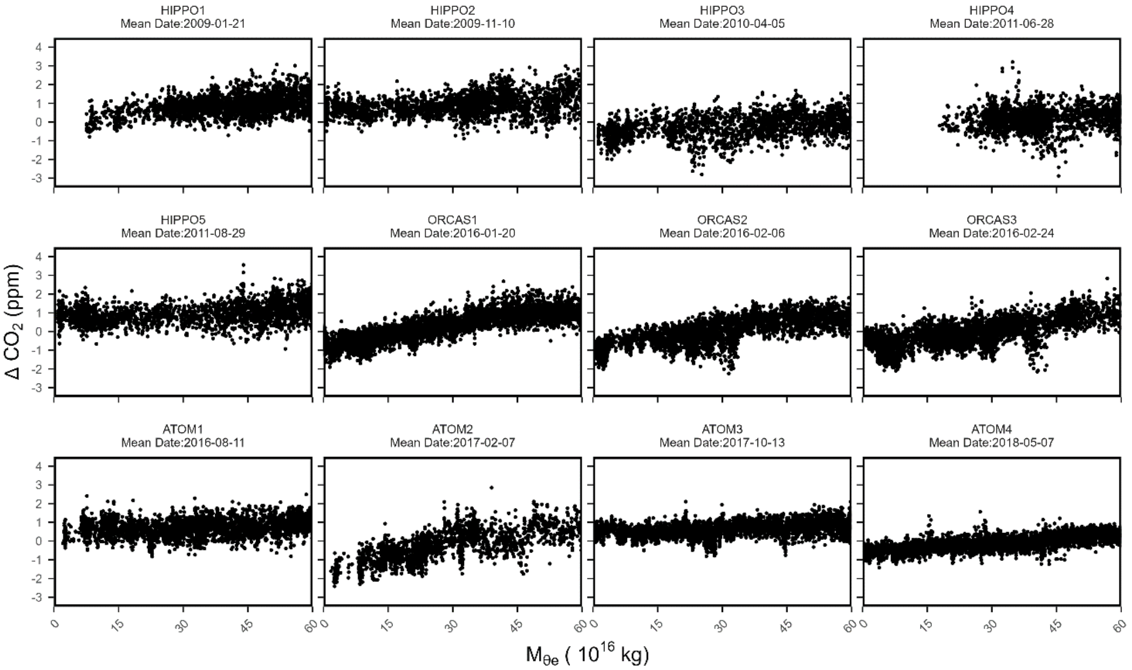

Fig. S15: Detrended airborne CO_2 observations (ΔCO_2) expressed on the $M_{\theta e}$ coordinate. We note that we have dense measurements across all $M_{\theta e}$ surfaces in each campaign except close-to-0 $M_{\theta e}$ during HIPPO1, ATom1, and ATom2. We also do not have measurements in the entire first $M_{\theta e}$ band during HIPPO4 (no observation lower than 17.88 $M_{\theta e}$).

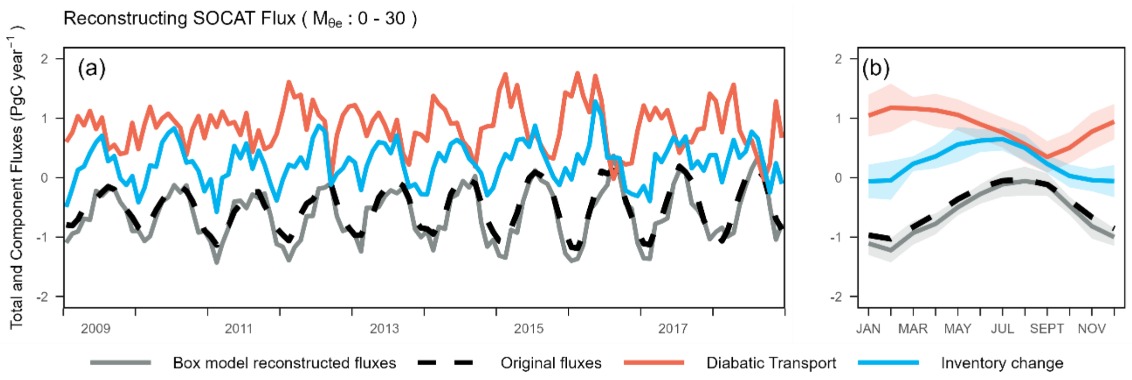

Fig. S16: Similar to Fig. 3, but reconstructing air-sea CO_2 flux from the neural-network interpolation of SOCAT data that is forward transported by the TM3 model, together with inverted fossil fuel CO_2 flux and ecosystem CO_2 flux from the Jena sEXTocNEEv2020. For this reconstruction, we use the diabatic mixing rates of the Jena sEXTocNEEv2020 (TM3 transport model) as in Fig. 2, rather than recalculating the mixing rates based on the flux field and atmospheric CO2 field. The comparison of the grey and black dashed curves shows that biases are small, with a small systematic offset in summer. The offset suggests that the mixing rates derived from simulated atmospheric CO_2 data depends slightly on the original CO_2 flux fields. This error is not directly relevant to our reported fluxes based on observations, which use MSE-based mixing rates.

Table S1: RMSE (PgC yr^{-1}) of reconstructed monthly surface CO_2 fluxes compared to the original fluxes.

M_{θ_e} band (10^{16} kg)	RMSE of each inversion products			
	Jena CO_2 inversion sEXTocNEET_v2020	CarbonTracker 2019b	CAMS V20r1	MIROC-ACTM2020
0-15	0.067	0.097	0.048	0.094
15-30	0.066	0.086	0.081	0.067
30-45	0.083	0.118	0.113	0.109

Table S2: Summary of research flight number, latitude coverage, and duration of each airborne mission in the Southern Hemisphere.

Campaign	Flight numbers	Latitude coverage	Date
HIPPO1	5 – 10	66.2°S – 0.0°S	2009.01.16 – 2009.01.28
HIPPO2	4 – 8	66.0°S – 0.0°S	2009.11.07 – 2009.11.16
HIPPO3	4 – 8	66.9°S – 0.0°S	2010.03.31 – 2010.04.10
HIPPO4	4 – 8	58.0°S – 0.0°S	2011.06.22 – 2011.07.03
HIPPO5	7 – 11	67.2°S – 0.0°S	2011.08.24 – 2011.09.03
ORCAS1	1 – 6	69.0°S – 33.3°S	2016.01.15 – 2016.01.25
ORCAS2	7 – 11	75.0°S – 35.0°S	2016.01.30 – 2016.02.12
ORCAS3	12 – 19	68.5°S – 18.3°S	2016.02.18 – 2016.02.29
ATom1	4 – 8	65.3°S – 0.0°S	2016.08.06 – 2016.08.17
ATom2	4 – 8	65.3°S – 0.0°S	2017.02.03 – 2017.02.15
ATom3	4 – 9	80.1°S – 0.0°S	2017.10.06 – 2017.10.19
ATom4	4 – 9	86.2°S – 0.0°S	2018.05.01 – 2018.05.14

Table S3: Atmospheric inversion products.

Product	Years	Transport Model	Resolution (lon x lat x vertical level)	Meteorology	Reference
Jena Inversion sEXTocNEET_v2020	1999-2019	TM3	4 x 5 x 19	NCEP	(23)
Carbon Tracker 2019b	2000-2018	TM5	3 x 2 x 25	ERA-Interim	(24)
CAMS v20r1	1979-2020	LMDZ6A	3.75 x 1.875 x 39	ERA5	(25–27)
MIROC-ACTM2020	1996-2019	MIROC4-ACTM	2.8 x 2.8 x 67	JRA-55	(28)

Table S4: Airborne-based air-sea CO_2 fluxes estimated for each campaign and $M_{\theta e}$ band. The mean day of year of each airborne campaign is also listed. Positive flux denotes net outgassing into the atmosphere. Latitudes represent mean annual locations of $M_{\theta e}$ boundaries. We did not resolve flux estimates in the first two bands of HIPPO4 because there is no observation data within the entire first $M_{\theta e}$ band (0-15 10^{16} kg).

Campaign	Day of year	M_{θ_e}(10^{16} kg): 0-15 Latitude: 90°S - 51°S		M_{θ_e}(10^{16} kg): 15-30 Latitude: 51°S - 43°S		M_{θ_e}(10^{16} kg): 30-45 Latitude: 43°S - 39°S		M_{θ_e}(10^{16} kg): 0-30 Latitude: 90°S - 43°S	
		Flux (PgC yr^{-1})	Uncertainty (PgC yr^{-1})	Flux (PgC yr^{-1})	Uncertainty (PgC yr^{-1})	Flux (PgC yr^{-1})	Uncertainty (PgC yr^{-1})	Flux (PgC yr^{-1})	Uncertainty (PgC yr^{-1})
HIPPO1	22	-0.68	0.24	-0.28	0.39	-0.01	0.50	-0.97	0.40
HIPPO2	314	-0.03	0.15	-0.35	0.42	-0.24	0.50	-0.39	0.46
HIPPO3	95	0.20	0.15	-0.54	0.46	-0.54	0.74	-0.34	0.47
HIPPO4	179	/	/	/	/	-0.61	1.48	/	/
HIPPO5	241	0.10	0.21	-0.02	0.63	-0.47	1.21	0.08	0.64
ORCAS1	20	-0.61	0.24	-0.66	0.24	-0.19	0.39	-1.27	0.32
ORCAS2	37	-0.50	0.24	-0.73	0.37	-0.25	0.36	-1.23	0.42
ORCAS3	55	-0.44	0.19	-0.45	0.25	-0.34	0.36	-0.90	0.28
ATom1	223	0.28	0.16	-0.15	0.30	-0.52	0.73	0.13	0.32
ATom2	40	-0.61	0.24	-0.99	0.41	-0.02	0.77	-1.61	0.46
ATom3	286	0.15	0.14	-0.46	0.28	-0.38	0.49	-0.31	0.33
ATom4	127	0.23	0.14	-0.30	0.34	-0.45	0.60	-0.07	0.38

Table S5: Atmospheric CO_2 gradients across $M_{\theta e}$ bands observed by each airborne campaign. The adjusted gradient is also shown here, which is calculated by subtracting the bias due to limited spatial coverage (detailed in *SI Appendix*, Text S1 and Fig. S12)

Campaign	$M_{\theta_e}^{15-30}$-$M_{\theta_e}^{0-15}$			$M_{\theta_e}^{30-45}$-$M_{\theta_e}^{15-30}$			$M_{\theta_e}^{45-60}$-$M_{\theta_e}^{30-45}$		
	Gradient (ppm)	Adjusted gradient (ppm)	Uncertainty (ppm)	Gradient (ppm)	Adjusted gradient (ppm)	Uncertainty (ppm)	Gradient (ppm)	Adjusted gradient (ppm)	Uncertainty (ppm)
HIPPO1	0.53	0.51	0.16	0.32	0.32	0.17	0.08	0.18	0.10
HIPPO2	0.09	0.03	0.11	0.23	0.20	0.17	0.14	0.27	0.10
HIPPO3	0.08	0.09	0.11	0.18	0.23	0.15	0.08	0.23	0.13
HIPPO4	/	/	/	0.08	0.12	0.21	0.08	0.16	0.13
HIPPO5	0.14	0.05	0.15	0.16	0.06	0.14	0.15	0.14	0.14
ORCAS1	0.71	0.60	0.09	0.66	0.64	0.10	0.20	0.47	0.08
ORCAS2	0.31	0.50	0.15	0.65	0.57	0.12	0.22	0.42	0.07
ORCAS3	0.38	0.53	0.09	0.59	0.42	0.08	0.39	0.44	0.07
ATom1	-0.01	-0.06	0.10	0.12	0.07	0.06	0.14	0.15	0.09
ATom2	0.68	0.47	0.16	0.52	0.62	0.15	0.31	0.32	0.19
ATom3	0.03	0.03	0.08	0.24	0.23	0.08	0.21	0.31	0.08
ATom4	0.29	0.22	0.05	0.13	0.22	0.08	0.14	0.21	0.06

Table S6: Bias of averaged CO_2 concentration due to limited spatial coverage (detailed in *SI Appendix,* Text S1). A positive value indicates that the limited spatial coverage would bias the observed average CO_2 of the corresponding $M_{\theta e}$ band too high. We also show the 1σ uncertainty of these corrections (± values), which are calculated as the standard deviation of the correction of four models for each campaign or sub-campaign and $M_{\theta e}$ band.

Campaign (sub-campaign)	M_{θ_e}(10^{16} kg) band			
	0-15	15-30	30-45	45-60
HIPPO1	0.01±0.06	0.02±0.09	0.02±0.06	-0.07±0.06
HIPPO2	0.00±0.09	0.06±0.02	0.09±0.04	-0.04±0.06
HIPPO3	0.23±0.08	0.22±0.03	0.18±0.02	0.03±0.06
HIPPO4	/	0.13±0.08	0.09±0.07	0.00±0.05
HIPPO5	0.03±0.03	0.12±0.07	0.21±0.07	0.23±0.09
ORCAS1	0.05±0.07	0.16±0.05	0.18±0.07	-0.09±0.01
ORCAS2	0.05±0.05	-0.14±0.11	-0.07±0.02	-0.26±0.04
ORCAS3	-0.06±0.05	-0.20±0.03	-0.03±0.04	0.02±0.04
ATom1	-0.02±0.06	0.02±0.01	0.07±0.01	0.06±0.08
ATom2	-0.21±0.07	0.00±0.11	-0.09±0.08	-0.11±0.15
ATom3	0.00±0.06	-0.01±0.04	0.00±0.04	-0.10±0.05
ATom4	0.07±0.02	0.13±0.03	0.14±0.05	0.07±0.02

Table S7: AR_{10-sec} coefficient (unitless) and 1σ uncertainty (ppm) of random error $\epsilon(t)$ estimated from the autoregressive model for each pair of in situ instruments (detailed in SI Appendix, Text S2.1). Here we only show the mean values for each campaign, averaged from all flights of the campaign. We note that the variability of parameters within a campaign is generally small.

Campaign (sub-campaign)	Instrument					
	AO2 - QCLS		AO2 - OMS		AO2 - NOAA Picarro	
	AR_{10-sec}	1σ of $\epsilon(t)$	AR_{10-sec}	1σ of $\epsilon(t)$	AR_{10-sec}	1σ of $\epsilon(t)$
HIPPO1	0.85	0.41	0.83	0.44		
HIPPO2	0.83	0.43	0.83	0.47		
HIPPO3	0.82	0.44	0.84	0.46		
HIPPO4	0.80	0.45	0.82	0.46		
HIPPO5	0.82	0.47	0.82	0.48		
ORCAS1	0.83	0.27			0.83	0.27
ORCAS2	0.85	0.30			0.83	0.26
ORCAS3	0.85	0.29			0.85	0.29
ATom1	0.77	0.47			0.74	0.47
ATom2	0.79	0.51			0.79	0.52
ATom3	0.84	0.28			0.83	0.27
ATom4	0.81	0.27			0.82	0.27

Table S8: 1σ Interannual variability (IAV, PgC yr^{-1}) of air-sea CO_2 flux for each $M_{\theta e}$ band and each airborne campaign or sub-campaign, as suggested by the MIROC-ACTM model. IAV is calculated as detailed in *SI Appendix*, Text S2.2.

Campaign	M_{θ_e} (10^{16} kg) band		
	0-15	15-30	30-45
HIPPO1	0.090	0.042	0.044
HIPPO2	0.062	0.032	0.023
HIPPO3	0.076	0.038	0.039
HIPPO4	/	0.049	0.032
HIPPO5	0.085	0.045	0.045
ORCAS1	0.091	0.043	0.045
ORCAS2	0.076	0.027	0.024
ORCAS3	0.071	0.024	0.023
ATom1	0.090	0.048	0.048
ATom2	0.077	0.026	0.023
ATom3	0.083	0.043	0.026
ATom4	0.064	0.037	0.041

References

1. Y. Jin, *et al.*, A mass-weighted atmospheric isentropic coordinate for mapping chemical tracers and computing inventories. *Atmos. Chem. Phys.* **21**, 217–238 (2021).
2. F. Elorrieta, S. Eyheramendy, W. Palma, Discrete-time autoregressive model for unequally spaced time-series observations. *Astron. Astrophys.* **627**, A120 (2019).
3. C. Sweeney, *et al.*, Seasonal climatology of CO_2 across North America from aircraft measurements in the NOAA/ESRL Global Greenhouse Gas Reference Network. *J. Geophys. Res. Atmos.* **120**, 5155–5190 (2015).
4. B. B. Stephens, *et al.*, Airborne measurements of oxygen concentration from the surface to the lower stratosphere and pole to pole. *Atmos. Meas. Tech.* **14**, 2543–2574 (2021).
5. T. Takahashi, *et al.*, Climatological mean and decadal change in surface ocean pCO_2, and net sea-air CO_2 flux over the global oceans. *Deep. Res. Part II Top. Stud. Oceanogr.* **56**, 554–577 (2009).
6. R. W. Reynolds, N. A. Rayner, T. M. Smith, D. C. Stokes, W. Wang, An improved in situ and satellite SST analysis for climate. *J. Clim.* **15**, 1609–1625 (2002).
7. L. L. Robbins, M. E. Hansen, J. A. Kleypas, S. C. Meylan, CO2calc: A user-friendly carbon calculator for Windows , Mac OS X , and iOS (iPhone). *U.S. Geol. Surv.*, 17 (2010).
8. R. Wanninkhof, Relationship between wind speed and gas exchange over the ocean revisited. *Limnol. Oceanogr. Methods* **97**, 7373–7382 (1992).
9. R. Gelaro, *et al.*, The Modern-Era Retrospective Analysis for Research and Applications, Version 2 (MERRA-2). *J. Clim.* **30**, 5419–5454 (2017).
10. M. A. Zondlo, M. E. Paige, S. M. Massick, J. A. Silver, Vertical cavity laser hygrometer for the National Science Foundation Gulfstream-V aircraft. *J. Geophys. Res. Atmos.* **115**, D20309 (2010).
11. S. G. Scott, T. P. Bui, K. R. Chan, S. W. Bowen, The meteorological measurement system on the NASA ER-2 aircraft. *J. Atmos. Ocean. Technol.* **7**, 525–540 (1990).
12. G. S. Diskin, J. R. Podolske, G. W. Sachse, T. A. Slate, Open-path airborne tunable diode laser hygrometer. *Diode Lasers Appl. Atmos. Sens.* **4817**, 196–204 (2002).
13. D. C. E. Bakker, *et al.*, An update to the surface ocean CO_2 atlas (SOCAT version 2). *Earth Syst. Sci. Data* **6**, 69–90 (2014).
14. C. Rödenbeck, *et al.*, Interannual sea-air CO_2 flux variability from an observation-driven ocean mixed-layer scheme. *Biogeosciences* **11**, 4599–4613 (2014).
15. K. S. Johnson, *et al.*, Biogeochemical sensor performance in the SOCCOM profiling float array. *J. Geophys. Res. Ocean.* **122**, 6416–6436 (2017).
16. P. Landschützer, N. Gruber, D. C. E. Bakker, U. Schuster, Recent variability of the global ocean carbon sink. *Global Biogeochem. Cycles* **28**, 927–949 (2014).

17. P. Landschützer, S. M. Bushinsky, A. R. Gray, A combined globally mapped carbon dioxide (CO_2) flux estimate based on the Surface Ocean CO_2 Atlas Database (SOCAT) and Southern Ocean Carbon and Climate Observations and Modeling (SOCCOM) biogeochemistry floats from 1982 to 2017 (NCEI Accession 0191304) (2019) https:/doi.org/10.25921/9hsn-xq82.

18. P. Landschützer, N. Gruber, D. C. E. Bakker, "An observation-based global monthly gridded sea surface pCO_2 product from 1982 onward and its monthly climatology (NCEI Accession 0160558). Version 5.5. NOAA National Centers for Environmental Information. Dataset." (2020) https:/doi.org/10.7289/v5z899n6.

19. S. M. Bushinsky, *et al.*, Reassessing Southern Ocean Air-Sea CO_2 Flux Estimates With the Addition of Biogeochemical Float Observations. *Global Biogeochem. Cycles* **33**, 1370–1388 (2019).

20. P. Friedlingstein, *et al.*, Global Carbon Budget 2020. *Earth Syst. Sci. Data* **12**, 3269–3340 (2020).

21. J. Hauck, *et al.*, Consistency and Challenges in the Ocean Carbon Sink Estimate for the Global Carbon Budget. *Front. Mar. Sci.* **7**, 571720 (2020).

22. M. C. Long, *et al.*, Strong Southern Ocean carbon uptake evident in airborne observations. *Science (80).* **374**, 1275–1280 (2021).

23. C. Rödenbeck, S. Houweling, M. Gloor, M. Heimann, CO_2 flux history 1982–2001 inferred from atmospheric data using a global inversion of atmospheric transport. *Atmos. Chem. Phys.* **3**, 1919–1964 (2003).

24. A. R. Jacobson, *et al.*, CarbonTracker CT2019B (2020) https:/doi.org/10.25925/20201008.

25. F. Chevallier, *et al.*, Inferring CO_2 sources and sinks from satellite observations: Method and application to TOVS data. *J. Geophys. Res. Atmos.* **110**, D24309 (2005).

26. F. Chevallier, On the parallelization of atmospheric inversions of CO_2 surface fluxes within a variational framework. *Geosci. Model Dev.* **6**, 783–790 (2013).

27. F. Chevallier, *et al.*, CO_2 surface fluxes at grid point scale estimated from a global 21 year reanalysis of atmospheric measurements. *J. Geophys. Res. Atmos.* **115**, D21307 (2010).

28. N. Chandra, *et al.*, Estimated regional CO_2 flux and uncertainty based on an ensemble of atmospheric CO_2 inversions. *Atmos. Chem. Phys.* **22**, 9215–9243 (2022).
